# Preventing Vaccine-Derived Poliovirus Emergence during the Polio Endgame

**DOI:** 10.1371/journal.ppat.1005728

**Published:** 2016-07-06

**Authors:** Margarita Pons-Salort, Cara C. Burns, Hil Lyons, Isobel M. Blake, Hamid Jafari, M. Steven Oberste, Olen M. Kew, Nicholas C. Grassly

**Affiliations:** 1 Department of Infectious Disease Epidemiology, St Mary’s Campus, Imperial College London, London, United Kingdom; 2 Division of Viral Diseases, Centers for Disease Control and Prevention, Atlanta, Georgia, United States of America; 3 Institute for Disease Modeling, Seattle, Washington, United States of America; 4 World Health Organization (WHO), Geneva, Switzerland; Institut Pasteur, FRANCE

## Abstract

Reversion and spread of vaccine-derived poliovirus (VDPV) to cause outbreaks of poliomyelitis is a rare outcome resulting from immunisation with the live-attenuated oral poliovirus vaccines (OPVs). Global withdrawal of all three OPV serotypes is therefore a key objective of the polio endgame strategic plan, starting with serotype 2 (OPV2) in April 2016. Supplementary immunisation activities (SIAs) with trivalent OPV (tOPV) in advance of this date could mitigate the risks of OPV2 withdrawal by increasing serotype-2 immunity, but may also create new serotype-2 VDPV (VDPV2). Here, we examine the risk factors for VDPV2 emergence and implications for the strategy of tOPV SIAs prior to OPV2 withdrawal. We first developed mathematical models of VDPV2 emergence and spread. We found that in settings with low routine immunisation coverage, the implementation of a single SIA increases the risk of VDPV2 emergence. If routine coverage is 20%, at least 3 SIAs are needed to bring that risk close to zero, and if SIA coverage is low or there are persistently “missed” groups, the risk remains high despite the implementation of multiple SIAs. We then analysed data from Nigeria on the 29 VDPV2 emergences that occurred during 2004−2014. Districts reporting the first case of poliomyelitis associated with a VDPV2 emergence were compared to districts with no VDPV2 emergence in the same 6-month period using conditional logistic regression. In agreement with the model results, the odds of VDPV2 emergence decreased with higher routine immunisation coverage (odds ratio 0.67 for a 10% absolute increase in coverage [95% confidence interval 0.55−0.82]). We also found that the probability of a VDPV2 emergence resulting in poliomyelitis in >1 child was significantly higher in districts with low serotype-2 population immunity. Our results support a strategy of focused tOPV SIAs before OPV2 withdrawal in areas at risk of VDPV2 emergence and in sufficient number to raise population immunity above the threshold permitting VDPV2 circulation. A failure to implement this risk-based approach could mean these SIAs actually increase the risk of VDPV2 emergence and spread.

## Author Summary

Global, coordinated withdrawal of serotype-2 OPV (OPV2) is planned for April 2016 and will mark a major milestone for the Global Polio Eradication Initiative (GPEI). Because OPV2 withdrawal will leave cohorts of young children susceptible to serotype-2 poliovirus, minimising the risk of new serotype-2 vaccine-derived poliovirus (VDPV2) emergences before and after OPV2 withdrawal is crucial to avoid large outbreaks. Supplementary immunisation activities (SIAs) with trivalent OPV (tOPV) could raise serotype-2 immunity in advance of OPV2 withdrawal, but may also create new VDPV2. To guide the GPEI strategy we examined the risks and benefits of implementing tOPV SIAs using mathematical models and analysis of data on the 29 independent VDPV2 emergences in Nigeria during 2004–2014. We found that in settings with low routine immunisation coverage, the implementation of a small number of tOPV SIAs could in fact increase the probability of VDPV2 emergence. This probability is greater if SIA coverage is poor or if there are persistently unvaccinated groups within the population. A strategy of tOPV SIA in sufficient number and with high coverage to achieve high population immunity in geographically-focused, at-risk areas is needed to reduce the global risk of VDPV2 emergence after OPV2 withdrawal.

## Introduction

Global and synchronous withdrawal of all live-attenuated oral poliovirus vaccines (OPV) is one of the major objectives of the global Polio Eradication & Endgame Strategic Plan 2013–2018 [1] and part of the global transition from OPV to inactivated poliovirus vaccine (IPV). Serotype 2 will be the first to be removed, with a planned date of April 2016. This means that trivalent OPV (tOPV) will be replaced by bivalent OPV (bOPV, containing Sabin virus types 1 and 3) in routine immunisation programmes, and tOPV will no longer be used in supplementary immunisation activities (SIAs). Furthermore, all OPV-using countries are recommended to introduce at least one dose of IPV in their routine immunisation programmes before the switch from tOPV to bOPV [2].

OPV use needs to be stopped because of its genetic instability. Attenuated vaccine (Sabin) polioviruses lose key genetic determinants of attenuation through mutation and/or recombination with other enterovirus serotypes during replication in the human gut [3]. In countries using OPV, approximately 1 child per 900,000 first OPV doses is estimated to develop vaccine-associated paralytic poliomyelitis (VAPP) [4]. The relative contributions of viral evolution (loss of key attenuating sites), immune function of the vaccine recipient and chance in the aetiology of VAPP are unclear. More significantly for the Global Polio Eradication Initiative (GPEI), vaccine polioviruses may spread from the recipient to his or her contacts, in rare cases leading to an outbreak of a vaccine-derived poliovirus (VDPV). VDPVs are defined as OPV-related isolates whose ~900-nucleotide sequence encoding the major capsid protein VP1 differs from that of the parental strain by >1% for serotypes 1 and 3, and >0.6% for serotype 2 [5]. VDPVs are classified into three categories: circulating VDPVs (cVDPVs), when there is evidence of person-to-person transmission; immunodeficiency-associated VDPVs (iVDPVs), shed by individuals with primary immunodeficiencies who have prolonged, sometimes chronic, virus excretion; and, ambiguous VDPVs (aVDPVs), which are isolates that cannot be classified as cVDPV or iVDPV despite thorough investigation [6,7]. Until July 2015, the definition of cVDPV required that genetically linked VDPVs were isolated from at least two AFP cases, but the GPEI now considers even single individual or environmental sample isolates to be cVDPV if their genetic features indicate prolonged circulation [7].

cVDPVs have transmission dynamics similar to wild polioviruses [8]. Since 2006, more than 680 acute flaccid paralysis (AFP) cases due to VDPVs have been reported worldwide [9], underlining the importance of VDPVs for the polio eradication endgame. Strikingly, >97% of those cases have been associated with serotype 2 [9], whose wild counterpart was last detected in 1999 [10]. The burden of serotype 2 VDPV (VDPV2) and the eradication of serotype 2 wild poliovirus (WPV2) in 1999 are the main motivations for the global withdrawal of serotype 2 OPV (OPV2) planned for April 2016.

Polioviruses spread where levels of immunity in the population are low and where environmental conditions such as sanitation and crowding facilitate virus transmission. As such, the detection of cVDPVs has historically been associated with poor population immunity [3,8,11–15]. However, the initial appearance of a VDPV in a population depends on different factors and the relationship with population immunity may be more complex. For example, the number of people infected with Sabin poliovirus, the duration of excretion among those infected, the extent of secondary transmission and the prevalence of other enteroviruses may all be important in determining the probability of VDPV emergence.

Worldwide OPV2 withdrawal will put the 155 countries currently using tOPV in their routine immunisation programmes at risk of outbreaks of VDPV2 given the associated increase in the number of children susceptible to that type. SIAs with tOPV prior to OPV2 withdrawal would increase population immunity to serotype 2 and have been proposed as a strategy to mitigate the risk of VDPV2 emergence and spread [16]. However, infrequent or poor-coverage SIAs could lead to limited immunity and potentially an adverse increase in risk resulting from poliovirus shedding and seeding of new VDPV. A better understanding of the factors associated with the risk of VDPV emergence and subsequent spread will help the GPEI to define a clear strategy on the number, timing and geographic extent of any tOPV SIA that minimises the risk of VDPV2 emergence at the time of and immediately after OPV2 withdrawal. Defining such a strategy is one of the priorities of the polio eradication program.

In this article, we first present mathematical models that describe the relationship between the coverage of routine and supplementary immunisation activities, and the probability of VDPV emergence and subsequent spread. To test the conclusions from the mathematical models, we then identified risk factors associated with past VDPV2 emergences in Nigeria. For this aim, we carried out a case-control analysis of those districts reporting the first case of poliomyelitis associated with each of the 29 independent VDPV2 emergences in Nigeria during 2004−2014 compared with districts without emergences. We also used logistic regression to identify the risk factors associated with the probability that a VDPV2 emergence resulted in >1 case of poliomyelitis. We finish by discussing the implications of our findings for the tOPV SIA strategy to reduce the risk of VDPV2 emergence during and post OPV2 withdrawal.

## Materials and Methods

### Mathematical models

The number of people infected with Sabin polioviruses is primarily determined by the number of doses of OPV administered during routine and supplementary immunisation activities and the level of population immunity. As the amount of OPV administered increases from zero, the number of Sabin-infected individuals will initially increase, but at some point further increases in OPV administration are likely to result in a decrease in the number of individuals infected because of the associated increase in the level of population immunity. This implies a trade-off in the levels of OPV use that will favour VDPV emergence. We developed two mathematical models to study this trade-off: an analytical model that includes only SIAs, and a more complex model that includes both routine immunisation and SIAs, which must be solved through numerical simulation. We used these models to investigate the risks and benefits of carrying out preventive campaigns with tOPV as a strategy to maximise population immunity to serotype 2 prior to OPV2 withdrawal.

#### Analytical model: SIAs only

We assume that a proportion of the population *s*_0_ is susceptible at the time that SIAs commence. We also assume that the SIAs occur at least 4 weeks apart but in sufficiently close succession such that we can ignore births and deaths during the period of analysis. Under these assumptions the expected proportion of children shedding vaccine poliovirus after a single SIA *i*_1_ can be written

$$i_{1}=y_{1}^{1}+y_{2}^{1}$$ (1)

where $y_{1}^{1}$ is the proportion shedding as a result of direct administration of OPV during the SIA and $y_{2}^{1}$ the proportion shedding as a result of secondary spread of OPV from that SIA (i.e. $y_{1}^{1}$ and $y_{2}^{1}$ differ on the source from which the infection is acquired, the vaccine or a Sabin-infected individual). We denote the coverage of the campaign by *v*, vaccine “take” by *w*, and the basic reproduction number of Sabin polioviruses by *R*_0*S*_. We assume all individuals who shed poliovirus following vaccine “take” are subsequently immune to reinfection and do not thereafter participate in poliovirus transmission. The expected proportion of children shedding following direct administration of OPV is then given by

$$y_{1}^{1}=s_{0}vw$$ (2)

assuming in this simple model that immunised individuals cannot be reinfected with OPV. The expected proportion of children who subsequently shed as a result of secondary transmission of OPV under a simple SIR model satisfies

$$y_{2}^{1}=\left(s_{0}-y_{1}^{1}\right)\left[1-\exp\left(-R_{0S}\left(y_{1}^{1}+y_{2}^{1}\right)\right)\right]$$ (3)

following Bailey 1975 [17], where $s_{0}-y_{1}^{1}$ is the proportion of children susceptible to infection. Note that secondary spread of OPV includes second, third and subsequent generation spread, and not only transmission from a vaccinated individual.

Following this SIA, the proportion of the population that is susceptible is reduced by *i*_1_ such that the fraction susceptible becomes *s*_1_ = *s*_0_−*i*_1_. We assume that all secondary transmission has finished before the next SIA. This is a reasonable assumption if *R*_0*S*_ < 1 because the majority of secondary transmission will take place in under 4 weeks (Section A.1.1 in S1 Text).

Subsequent SIAs are assumed to reach the same proportion *v* of the population (i.e. same coverage), which may either consist of randomly chosen individuals at each round (random coverage) or repeatedly reach the same fixed group of individuals (fixed coverage), meaning there is a persistently “missed” group. Each of the subsequent *n* SIAs will result in the proportions *i*_2_,*i*_3_,…,*i*_*n*_ of individuals shedding Sabin poliovirus, and *s*_2_,*s*_3_,…,*s*_*n*_ susceptible (see Section A.1.2 in S1 Text for modelling details under the two types of SIA coverage, random and fixed).

The number *r* of emergent VDPVs following the *n* SIAs can be considered a function of the number of individuals infected with Sabin poliovirus. The probability of Sabin reversion to VDPV may differ by whether the infection is primary or secondarily transmitted. In the simplest case, we assume all Sabin infections have the same, very small probability of reverting to a VDPV. In this case, the number of emergent VDPVs is given by

$$r≃\rho N\sum_{k=1}^{n}i_{k}$$ (4)

where *N* is the total population size and *ρ* is an unknown parameter that determines the absolute risk of VDPV emergence (i.e. reversion of a Sabin virus). The latter is assumed to capture unknown risk factors such as the prevalence of other enterovirus serotypes that could act as partners for recombination and the prevalence of primary immunodeficiencies that might result in prolonged excretion of vaccine poliovirus. Because the number of emergent VDPVs depends on *ρ* and *N* via their product, we can reparameterize *r* by introducing the parameter *σ* = *ρN*:

$$r≃\sigma \sum_{k=1}^{n}i_{k}$$ (5)

The probability that an emergent VDPV spreads and produces one or more AFP cases will depend on the size of the resulting outbreak. The cVDPVs characterised so far appear to show similar attack rates to wild poliovirus [8,12] and are therefore likely to have a reproduction number *R*_0*V*_ sufficient to result in a significant outbreak unless population immunity levels are high. The probability of an outbreak of VDPV for a simple epidemic model is given by *q* = max(1−1/(*R*_0*V*_ *s*),0), where *s* is the proportion of the population susceptible to infection. For a major outbreak, the duration of transmission will significantly exceed 4 weeks (unlike Sabin virus) and therefore we make the assumption that *s* ≈ *s*_*n*_, i.e. population susceptibility is defined at the end of all *n* SIAs. Assuming the overall risk of emergence is relatively small, this means that the number of emergent VDPVs that will produce an outbreak will be approximately Poisson distributed with mean proportional to *rq*. The probability of (at least) one outbreak of VDPV occurring is therefore given by

P(VDPV outbreak)=1−exp(−rq) (6)

and the expected proportion of individuals that have been infected with a VDPV once the outbreak has finished can be obtained using the equation for $y_{2}^{1}$ and replacing *R*_0*S*_ by *R*_0*V*_, $s_{0}-y_{1}^{1}$ by *s*_*n*_ and $y_{1}^{1}$ by $\rho \sum_{k=1}^{n}i_{k}$. Fig A in S1 Text illustrates the processes captured by this model.

We use this model to explore how the probability of a VDPV outbreak changes for different number of SIAs and different SIA coverage in a scenario without routine immunisation.

The model of Sabin virus spread during the *n* SIAs described above can be reformulated (Section A.2.1 in S1 Text) and using this equivalent version, some system’s properties can be studied analytically, leading to a more general understanding of the model’s behaviour. Very briefly, we show that multiple rounds of SIAs with the same coverage *v* are equivalent (in terms of the proportion of individuals infected with Sabin at some point and the proportion who remains susceptible after the SIAs) to a single round of SIA with vaccine coverage given as a certain function of *v*, *w* and *n*. For interested readers, the equivalent version of the model and the analytical results that can be obtained are given in Section A.2 in S1 Text.

#### Stochastic compartmental model: routine immunisation and SIAs

We wish to study the risk of VDPV emergence and spread in the context of OPV withdrawal. It is thus necessary to extend our previous conceptual framework to include routine immunisation. In order to include both routine and supplementary immunisation, we constructed a stochastic compartmental model of Sabin virus and VDPV spread that also considers births and deaths.

If a fixed proportion of individuals *v*_*ri*_ is reached by routine immunisation at each scheduled dose, and three doses of OPV are given, then assuming that only a fraction *w* of those doses will “take”, the proportion *c* of children who received the three doses and shed Sabin virus is *c* = *v*_*ri*_ *w*(1+(1−*w*)+(1−*w*)^2^). We model secondary spread of OPV through an SIR model with demography where the proportion *c* of children vaccinated and shedding the virus enter the model as infected with Sabin poliovirus, and the other children enter the model as susceptible. As before, a small proportion *ρ* of incident Sabin poliovirus infections are assumed to revert to VDPV, which may then spread in the population. In this simple case, we assume that this proportion is fixed and independent of whether infection was acquired directly through immunisation or through secondary spread of OPV. See Section A.3 in S1 Text for the full model description. As for the analytical model above, two versions of the stochastic model were implemented: one where each SIA reaches a fixed proportion of the population consisting of randomly chosen individuals at each round (random coverage, Table A in S1 Text), and one where the same individuals are reached at each SIA, thus leaving a persistently “missed” group (fixed coverage, Table B in S1 Text).

**Fig 1:**
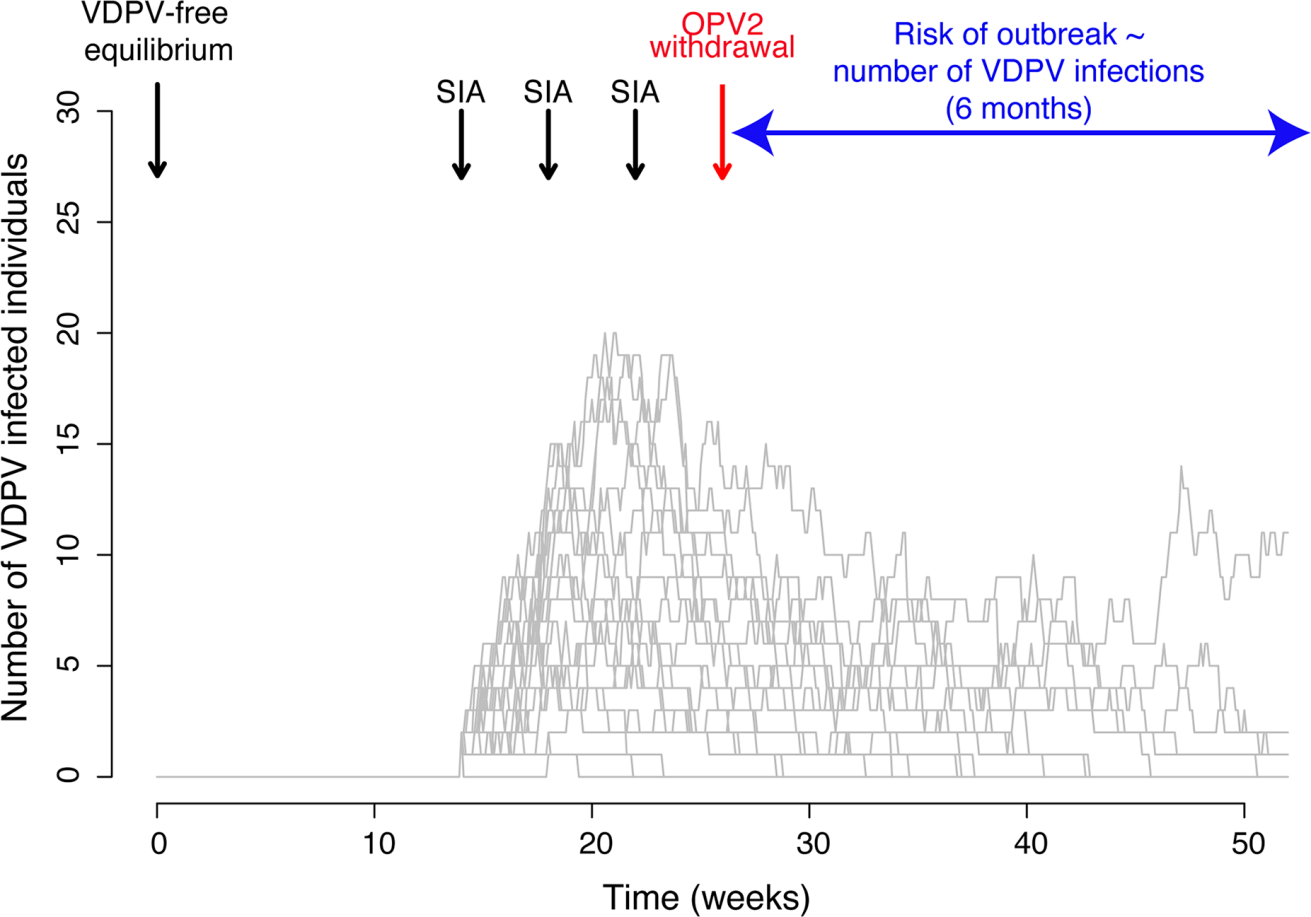
Illustration of results from the stochastic dynamic mathematical model of VDPV2 emergence and spread. The model is simulated for 1 year and a population of 10,000 individuals, starting from a VDPV-free equilibrium that includes routine immunisation. OPV2 withdrawal occurs at 6 months (red arrow) and the last tOPV SIA is assumed to occur 4 weeks before this date in agreement with current plans. SIAs are implemented 4 weeks apart. We define the risk of a VDPV2 outbreak after OPV2 withdrawal as the probability of having >200 incident VDPV2 infections during the 6 months following OPV2 cessation. In this illustration, the grey lines represent the number of VDPV infected individuals over time for 20 different simulations of the model assuming 20% routine immunisation coverage and 3 tOPV SIAs with 80% coverage implemented before OPV2 withdrawal.

We used the model to explore how the risk of VDPV2 emergence and spread varies depending on tOPV use in a context of OPV2 withdrawal and considering a population of 10,000 individuals. We simulated the model from a VDPV2-free initial equilibrium for 1 year under different scenarios that included different levels of routine immunisation coverage and between 0 and 5 SIAs (Fig 1). Routine immunisation with tOPV was assumed to stop at 6 months and the last tOPV SIA 4 weeks before this date corresponding to plans for SIAs with tOPV before OPV2 withdrawal. We defined the risk of a VDPV2 outbreak after OPV2 withdrawal as the probability of having >200 incident VDPV2 infections during the 6 months following OPV2 withdrawal (robustness of this threshold for increasing population size is explored, Fig F in S1 Text). This corresponds to a probability of approximately 20% of observing a case of poliomyelitis, given a case-to-infection ratio for serotype 2 of approximately 1:800 (based on data indicating 4–5 times lower pathogenicity for this serotype compared with serotype 1 [18], which has a case-to-infection ratio of about 1:150 [19,20]). We performed 500 simulations for each scenario and present the proportion of simulations that resulted in this outcome.

#### Parameter values

For both models (the SIA-analytical and the stochastic dynamic), the probability of vaccine “take” was set at *w* = 0.55 [21] and the reproduction number of Sabin virus was assumed to be <1 and fixed to *R*_0*S*_ = 0.5. The reproduction number of VDPVs was set at *R*_0*V*_ = 5, similar to estimated values for wild poliovirus [22]. Finally, the probability of Sabin virus reversion to VDPVs was set at *ρ* = 5×10^−4^, although we explored sensitivity of our results to this value.

### Risk factors for VDPV2 emergence and spread in Nigeria (2004−2014)

#### Data

Multiple emergences of VDPV2 were identified in Nigeria during 2004−2014 on the basis of detection of virus in stool collected from children with AFP and phylogenetic analysis of the P1/capsid region [15,23]. We recorded the district of residence and date of onset of paralysis for the first case of AFP associated with each of these independent emergences.

We created a database for all the districts of Nigeria for the period 2004−2014 that included variables describing routine immunisation coverage, serotype-2 population immunity, the number of tOPV SIAs in the preceding 6 months (since VDPV2 emergence will precede detection in a child with AFP by about this time period, based on observed genetic divergence from the Sabin virus [23,24]), and demographic variables including mean household size, population density and annual number of births (Section B in S1 Text). We compiled the data for 6-month periods, defined as April-September and October-March (roughly corresponding with a high-transmission period during spring-summer months and a low-transmission period in autumn-winter months). This resulted in 772x19 = 14,668 district 6-month observations (from April 2004 to September 2014). Serotype-2 population immunity was estimated among children 0–2 years old based on the vaccination histories of children with non-polio AFP and the SIA calendar (Section C in S1 Text). Routine immunisation coverage was estimated interpolating data across the whole country for three doses of diphtheria-tetanus-pertussis (DTP3) vaccination from the Demographic and Health Surveys (DHS) [25] clusters (Section D in S1 Text).

#### Statistical analyses

We used a “case-control” approach to compare districts in a given 6-month period where VDPV2 emergences occurred with districts in a given 6-month period where there were no VDPV2 emergences. In this framework, a “case” was defined as a district over a 6-month period (district−6-months) where the first AFP case associated with a VDPV2 emergence was detected. A “control” was defined as a district−6-months with no VDPV2 emergences. Each district−6-months case was matched to 20 district−6-months controls from the same 6-month period to allow for potential confounding as a result of secular trends. The controls were randomly selected among all the district−6-months candidates that satisfied the matching criteria (i.e. same 6-month period).

We performed univariable and multivariable analyses using conditional logistic regression models. Odds ratios (ORs) and 95% confidence intervals (CIs) were calculated for all explanatory variables and associations with *P* values <0.05 were considered to be statistically significant. All variables with Wald test *P* <0.05 in univariable analyses were included in the multivariable models and the final multivariable model was the one with the lowest AIC [26]. All the analyses were performed using the “survival” package [27] from R [28].

Finally, we also used univariable logistic regression analyses to test whether any of the variables were associated with the probability that a VDPV2 emergence resulted in >1 case of poliomyelitis (i.e. established a circulating lineage), thus corresponding to the definition of cVDPV used until July 2015, which required that genetically linked VDPVs were isolated from at least two AFP cases.

## Results

### Mathematical models

**Fig 2:**
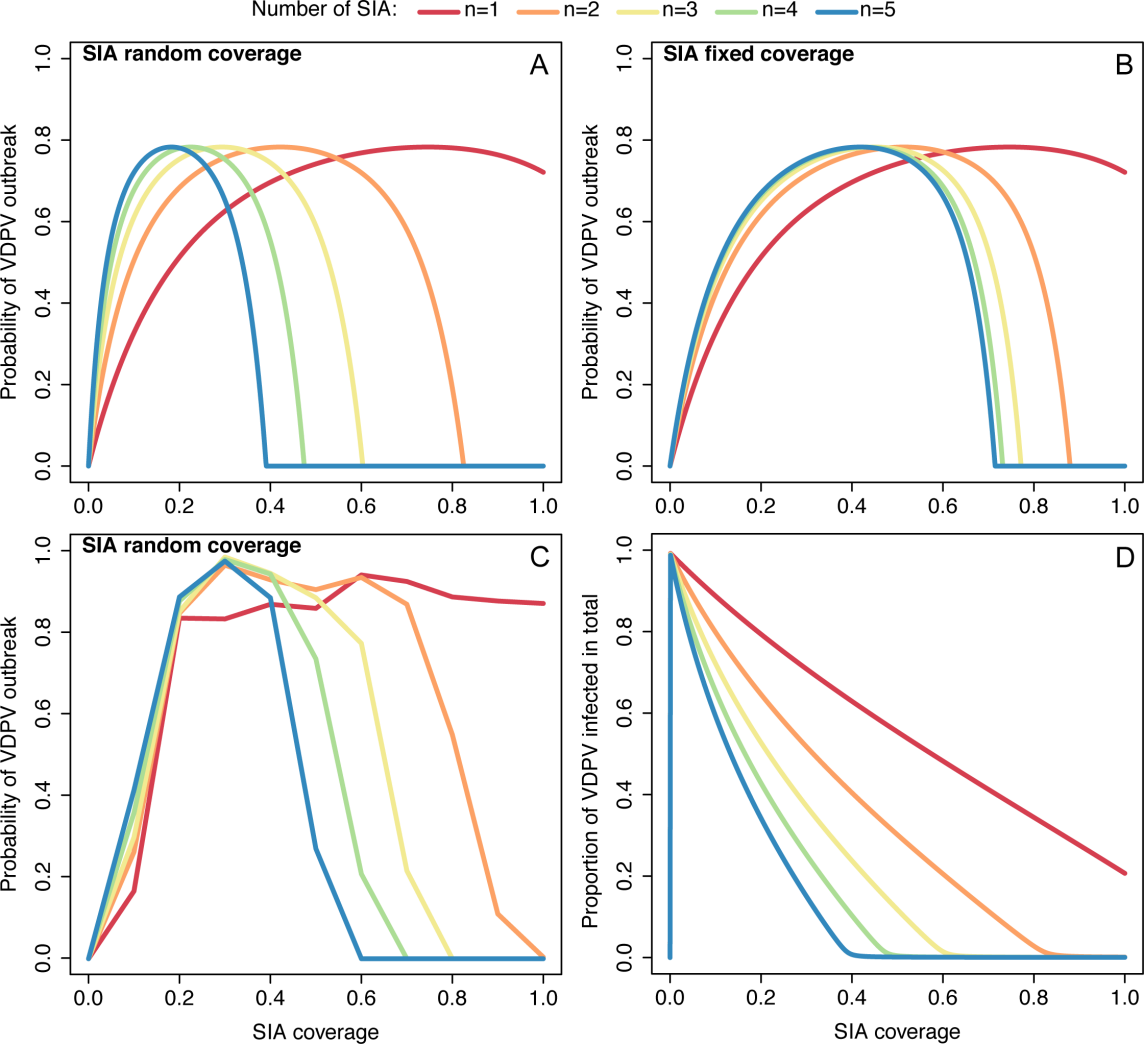
Probability of VDPV outbreak as a function of SIA coverage for scenarios with no routine immunisation. (A) Results for the analytical model assuming that each SIA reaches the same proportion of the population consisting of randomly chosen individuals at each round (SIA random coverage). (B) Results for the analytical model assuming that SIAs reach the same individuals at each round (SIA fixed coverage) with secondary spread of Sabin virus to the “missed” group. (C) Results for the stochastic dynamic model assuming SIA random coverage. (D) Proportion of individuals infected with VDPV (outbreak size) for the analytical model assuming SIA random coverage. The colours correspond to different number of SIAs. The simulations assume *σ* = 5, which corresponds for example to a population of 10,000 individuals and a probability of Sabin reversion to VDPV of *ρ* = 5×10^−4^. A sensitivity analysis to the value of *σ* is provided in Fig B in S1 Text.

We explored the probability of a VDPV outbreak for different numbers of supplementary campaigns in a scenario without routine immunisation and considering a population completely susceptible (Fig 2). Using the analytical model, if the individuals reached at each campaign are randomly chosen (assuming the same coverage at each campaign), the risk of a VDPV outbreak is maximised at low and intermediate levels of SIA coverage (Fig 2A). The exact location of the peak in risk depends on the number of SIAs, rapidly shifting to lower values of SIA coverage for increasing number of SIAs. In particular, for a single SIA with 100% coverage, the probability of a VDPV outbreak is around 70%, which is explained by the relatively small proportion of children that will be protected after the campaign, due to the limited (~50%) immunogenicity of OPV. As expected, the size of any resulting outbreak is also significantly smaller for increasing number of SIAs (Fig 2D). Although the absolute risk of VDPV emergence depends on the assumed probability of reversion of Sabin poliovirus to a VDPV (*ρ*) and the assumed population size (*N*) via *σ* = *ρN*, the location of the peak in risk does not change unless the value of *σ* is so low or high as to make VDPV emergence impossible or inevitable respectively. We provide a sensitivity analysis of the probability of VDPV outbreak to the value of *σ* in Fig B in S1 Text. In particular, when *σ* becomes sufficiently large, the probability of a VDPV outbreak becomes a stepwise function of SIA coverage (Fig B in S1 Text).

If the same individuals are reached at each campaign, thus leaving a “missed” group that is only immunised through secondary spread of Sabin virus, the risk of a VDPV outbreak is maximised at intermediate levels of SIA coverage. More importantly, increasing the number of SIAs above 4 barely reduces the risk, which becomes zero only above 70% coverage after 4 or more SIAs (Fig 2B). In other words, there is a threshold in SIA coverage under which the risk does not decrease despite increasing the number of campaigns. The existence of this threshold can be shown analytically (Section A.2.3 in S1 Text), and for both random and fixed coverage, an expression for the minimum SIA vaccine coverage required to have zero probability of outbreak can be found (Section A.2.3 in S1 Text). A sensitivity analysis of the minimum SIA coverage required for zero probability of a VDPV outbreak to a broad range of values of the reproduction number of Sabin virus and the reproduction number of VDPVs is shown in Figs D and E in S1 Text. As expected, the minimum SIA coverage to bring the probability of a VDPV outbreak to zero increases for increasing values of the reproduction number of VDPVs, however, it slightly decreases for increasing values of the reproduction number of Sabin virus, because of the associated increase in the number of individuals who will be immunised by secondary spread of OPV from vaccinees.

The risk of observing an outbreak of VDPV obtained with the more complex model in the absence of routine immunisation coverage displays a shape similar to that obtained with the analytical model, although stochastic extinction results in a lower risk at low values of SIA coverage (Fig 2C).

**Fig 3:**
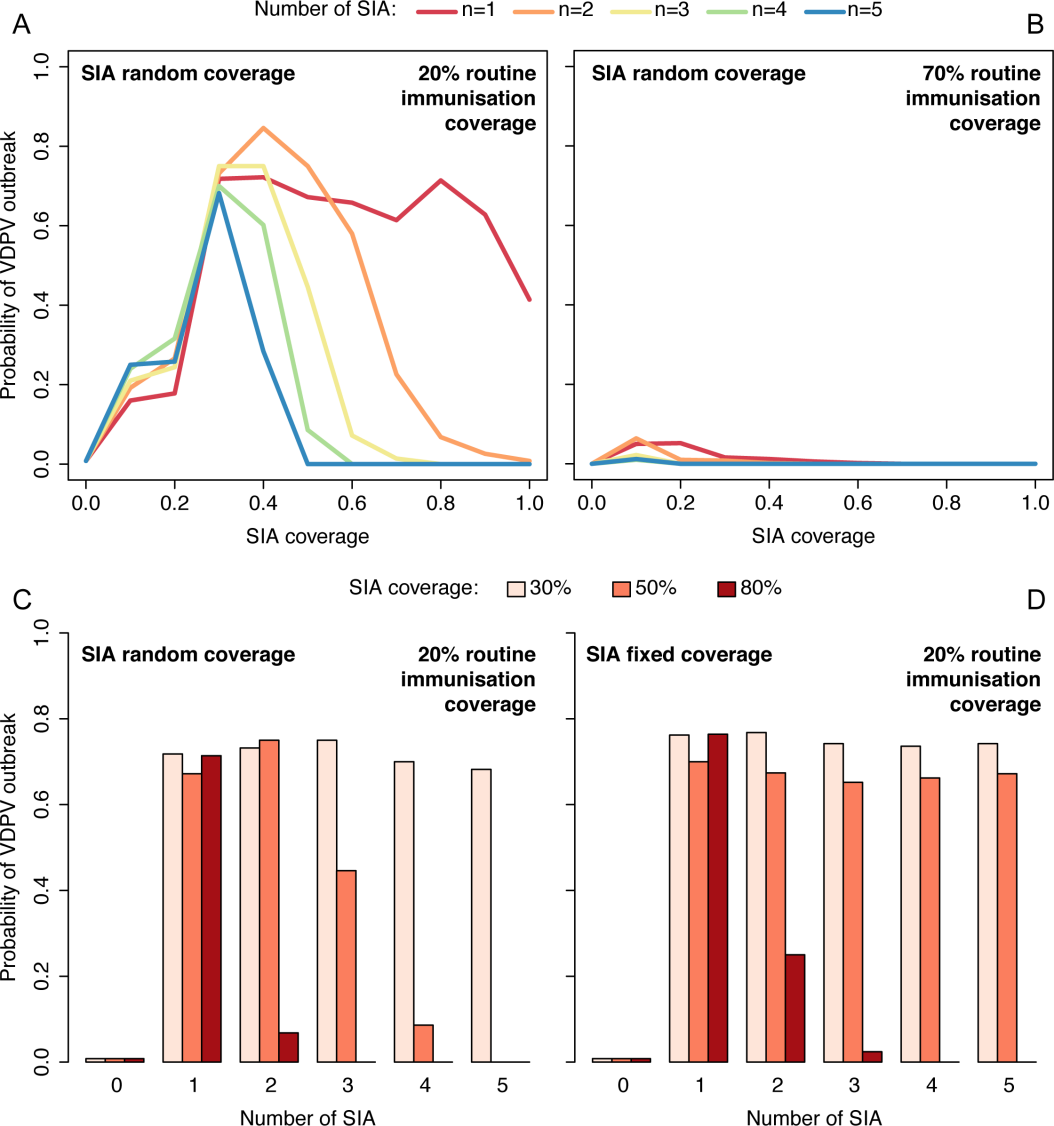
Probability of VDPV outbreak for scenarios with both routine immunisation and SIAs. Probability of a VDPV outbreak during the 6 months following OPV withdrawal as a function of SIA coverage for the stochastic dynamic model with routine immunisation and SIA random coverage, assuming (A) 20% and (B) 70% routine immunisation coverage. Probability of a VDPV outbreak in a setting with 20% routine immunisation coverage as a function of the number of SIAs, assuming they reach a fixed proportion of the population at each round consisting of (C) randomly chosen individuals each time (SIA random coverage) or (D) the same individuals each time such that there is a persistently “missed” group (SIA fixed coverage). The probabilities are based on 500 simulations of the model.

The stochastic SIR model allows the study of the risk of VDPV2 outbreak in the context of OPV2 withdrawal. Including reasonable levels of routine immunisation coverage results in a significant reduction in the risk of VDPV2 outbreaks during the 6 months that follow OPV2 withdrawal (Fig 3A and 3B). This risk becomes almost negligible when routine coverage is high (Fig 3B), but for low levels of routine immunisation coverage, multiple tOPV SIAs preceding OPV2 withdrawal are needed to avoid seeding new VDPV2 (Fig 3C and 3D). Notably, in the context of low routine immunisation coverage, a single SIA seems to highly increase the risk irrespective of SIA coverage, and at least 3 campaigns at high coverage are needed to bring that risk close to zero (Fig 3C and 3D). If campaign coverage is only intermediate and there is a persistently “missed” group (i.e. SIAs reach the same individuals at each round), the risk of VDPV2 outbreak remains high even after 4 or 5 SIAs (Fig 3D).

### Risk factors for VDPV2 emergence and spread in Nigeria (2004−2014)

**Fig 4:**
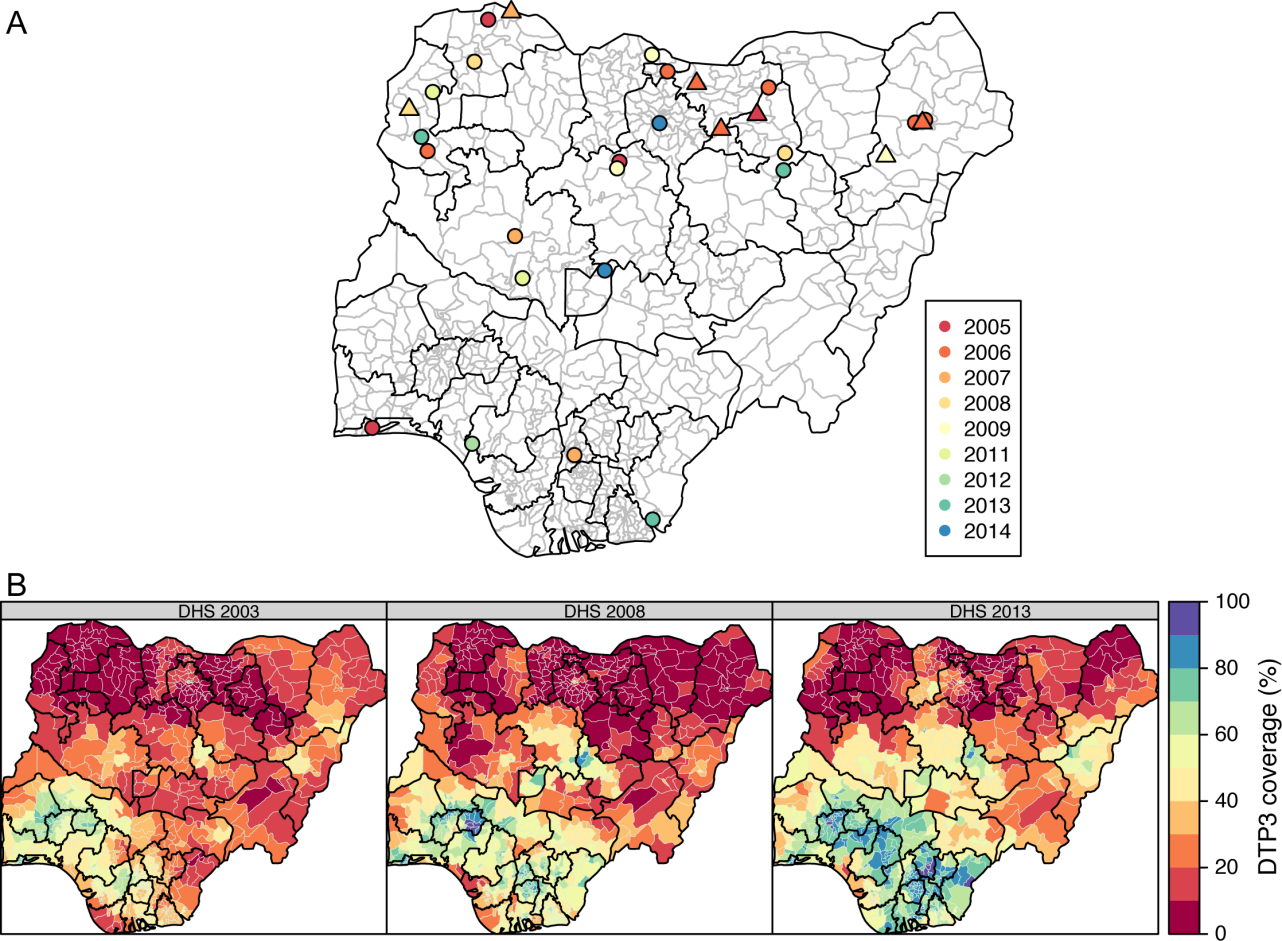
Independent VDPV2 emergences in Nigeria (2004−2014). (A) Time and geographical distribution of the first AFP case associated with each of the 29 independent VDPV2 emergences in Nigeria during 2004−2014. Triangles represent isolates that established circulating lineages (>1 case of poliomyelitis), whereas points represent single isolates. The triangles and points representing the emergence events are randomly plotted within the corresponding districts. The colours correspond to the year of onset of paralysis. (B) Maps of the predicted DTP3 coverage by district for the 2003, 2008 and 2013 DHS. The publication of this map does not imply the expression of any opinion whatsoever on the part of WHO concerning the legal status of any territory, city or area or of its authorities, or concerning the delimitation of its frontiers or boundaries.

A total of 29 independent VDPV2 emergence events were identified in Nigeria during the study period, of which 7 resulted in more than one case of poliomyelitis (Fig 4A, Table C in S1 Text). This resulted in 28 cases in the case-control analysis, since two emergences took place in the same district during the same 6-month period (Maiduguri, Borno state, between April and September 2006). The 28 cases were matched to 560 controls.

The number of tOPV SIAs in the previous 6 months varied between 0 and 5 (Fig H in S1 Text). Important changes over time occurred, due to a progressive and rapid removal of tOPV from SIA since 2006, which was replaced by bOPV, mOPV1 and mOPV3 [29]. tOPV was re-introduced in SIAs since mid-2009. These changes over time were also reflected in estimated serotype-2 immunity among children 0–2 years old, which reached very low levels in 2008 and 2009, and increased again from 2010 onwards (Fig G in S1 Text). In general, serotype-2 population immunity was higher in Southern districts. Routine immunisation coverage was also higher in the South and increased during the study period (Fig 4B).

The annual number of births per district was highly variable, ranging from 42 children (Bakassi, Cross River state) to 57,710 children (Alimosho, Lagos state), with a median of 7,542 (Fig I in S1 Text). Population density was also highly variable, ranging between an average of 9.37 (Teungo, Adamawa state) and 55,450 people per km^2^ (Ajeromi-Ifelodun, Lagos state), with a median of 218.70 (Fig J in S1 Text). The mean number of household members per district remained nearly constant over the study period, ranging between 3.26 and 6.31, and displayed a North-South gradient (Fig Q in S1 Text).

In the univariable analyses, a number of variables were associated with cases of VDPV2 emergence: (i) geographic region (North vs. South), (ii) serotype-2 population immunity, (iii) routine immunisation coverage, (iv) number of tOPV campaigns in the previous 6 months, (v) number of months since the last tOPV campaign, (vi) number of births, and (vii) number of household members (Table 1). Districts in the North had an increased risk of VDPV2 emergence compared to the South (odds ratio 5.52, [95% confidence interval 1.89−16.16]). This association may well reflect the existence of a North-South gradient in Nigeria for many demographic, social and economic variables [30]. The number of births was also associated with cases of VDPV2 emergence as it is a proxy for the size of the population exposed to OPV. Interestingly, among the variables related to OPV use, increased population immunity, routine immunisation coverage and the number of months since the last tOPV SIA were associated with a reduced odds of VDPV2 emergence. However, the number of campaigns in the previous 6 months was associated with an increase in the odds of VDPV2 emergence (Table 1).

**Table 1:** Univariable and multivariable analyses of cases (district−6-months where the first AFP case associated with a VDPV2 emergence was detected) vs. controls (district−6-months with no VDPV2 emergence). Abbreviations: CI, confidence interval; OR, odds ratio; *P*, p-value. Statistically significant p-values <0.05 are shown in boldface. Borderline significant p-values <0.1 are shown in italics.

	Univariable analyses			Best model (multivariable)		
				AIC = 146.63		
Variable	OR	(95% CI)	*P*	OR	(95% CI)	*P*
North vs. South	5.52	(1.89,16.16)	**0.002**			
Serotype-2 population immunity^a^	0.68	(0.56,0.83)	**<0.001**			
Routine immunisation coverage^b^	0.67	(0.55,0.82)	**<0.001**	0.69	(0.57,0.84)	**<0.001**
No. tOPV SIAs in the previous 6 months	2.97	(1.12,7.87)	**0.028**			
No. months since last tOPV SIA	0.92	(0.83,1.01)	*0*.*092*			
Annual No. births (log10)	13.09	(2.68,63.83)	**0.001**	16.81	(2.41,117.29)	**0.004**
Population density (log10)	0.94	(0.49,1.81)	0.858			
Mean number of household members	2.99	(1.65,5.46)	**<0.001**			

^a^ Odds ratio for a 10% absolute increase in serotype-2 population immunity.

^b^ Odds ratio for a 10% absolute increase in routine immunisation coverage.

The best multivariable model (lowest AIC, 146.63) retained two variables statistically significantly associated with cases of VDPV2 emergence: routine immunisation coverage and the annual number of births (Table 1). In this model, an absolute increase of 10% in routine immunisation coverage was estimated to reduce the odds of VDPV2 emergence by 31%. Adding the number of tOPV SIAs in the previous 6 months to the best model gave a very similar AIC (148.08), but the variable was not statistically significant (odds ratio 1.54, [95% confidence interval 0.47−5.07]).

**Fig 5:**
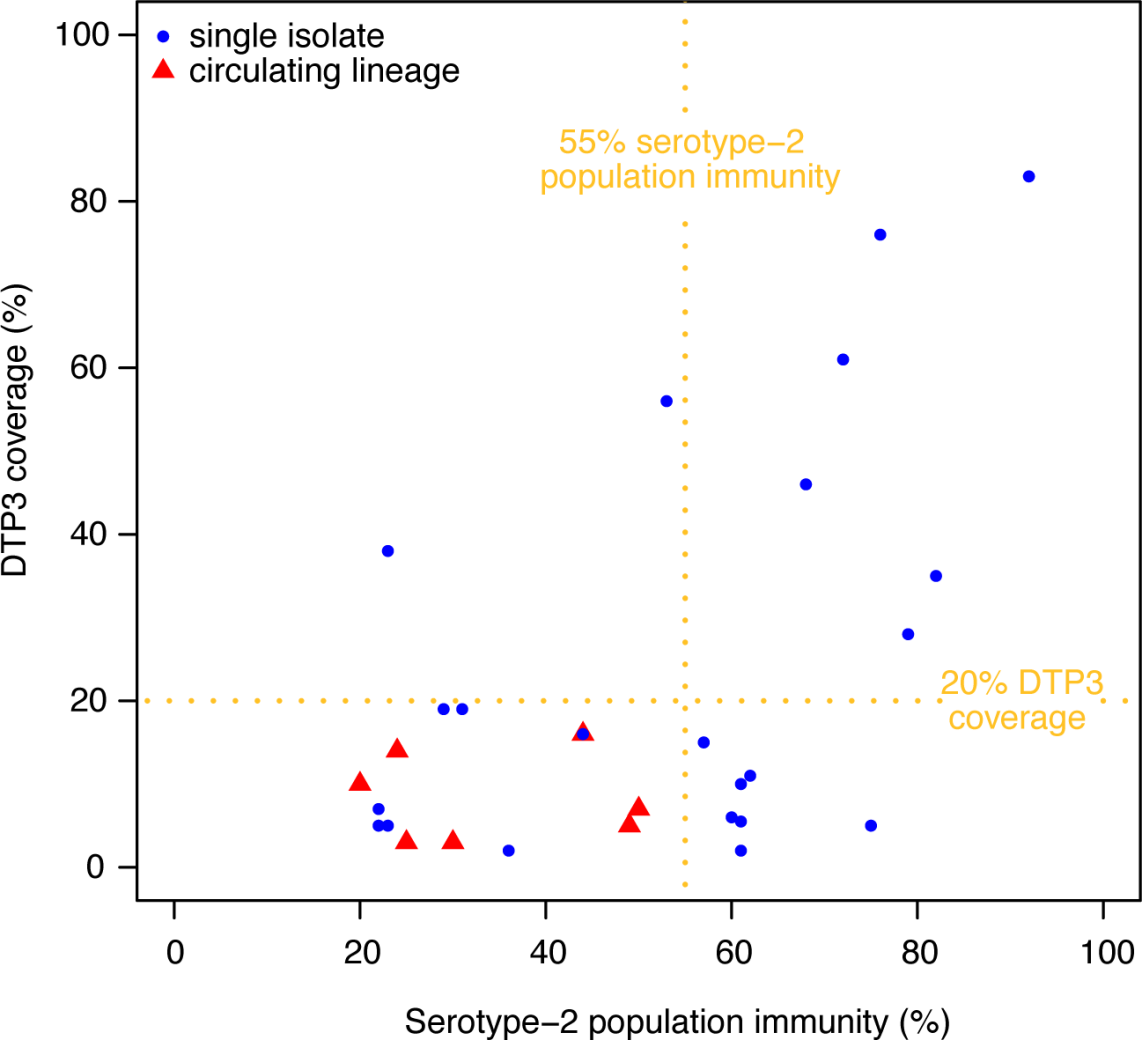
Risk factors for an emergent VDPV2 to establish a circulating lineage associated with >1 case of poliomyelitis in Nigeria. Serotype-2 population immunity and DTP3 coverage in districts in the 6-month period when the first AFP case associated with each of the 29 independent VDPV2 emergences in Nigeria during 2004−2014 were reported. Red triangles represent isolates that established circulating lineages, whereas blue circles represent single isolates.

The seven VDPV2 emergences that established circulating lineages (>1 case of poliomyelitis) occurred in districts with low to middle serotype-2 population immunity (<55%) and low routine immunisation coverage (<20%) (Fig 5). A univariable logistic regression analysis found that the probability of an emergent VDPV2 to establish a circulating lineage decreased for higher serotype-2 population immunity (p = 0.051). The other variables did not show a statistically significant association with the probability of a VDPV2 to establish a circulating lineage.

## Discussion

This study presents an analysis of the risk factors associated with the emergence and spread of VDPV, and provides a basis for strategic decisions about the optimal extent and number of mass campaigns with OPV in advance of OPV withdrawal.

First, using two simple mathematical models, we describe a trade-off between OPV use and the risk of VDPV emergence. Our findings indicate that immunity provided through routine immunisation counterbalances well the risk of VDPV emergence and spread. However, we found that in settings where routine immunisation coverage or the baseline level of population immunity is low, a small number of SIA campaigns could increase the risk of VDPV emergence compared to no campaigns. This is partly due to the low immunogenicity of OPV that makes necessary a certain number of SIA rounds to increase population immunity to levels that counterbalance the risk of seeding new VDPV through those campaigns. For example, our model predicted that in a setting with just 20% routine coverage with three tOPV doses (e.g. many districts in northern Nigeria), a single OPV SIA increased the risk of VDPV outbreak irrespective of SIA coverage (Fig 3). To bring population immunity to levels that completely counterbalanced the risk of VDPV outbreak, at least three rounds of supplementary campaigns at 80% coverage were needed. If only intermediate levels of campaign coverage were attained and SIAs persistently reached the same population leaving a persistently “missed” group, the risk of VDPV emergence remained high even when a high number of campaigns were implemented (Fig 3D). The existence of this threshold in SIA coverage under which the risk cannot decrease despite an increasing number of SIA illustrates how groups of unvaccinated children may hamper the efforts to minimise the risk of VDPV after OPV withdrawal.

Second, we identified risk factors associated with VDPV2 emergence and subsequent spread in Nigeria using epidemiologic, virologic and demographic data for 2004−2014. In both univariable and multivariable analyses, districts reporting the first case of poliomyelitis associated with a given VDPV2 emergence were more likely to have low routine immunisation coverage and a higher number of births. These districts were also more likely to have had a higher number of tOPV SIA in the previous 6 months, although this association was not statistically significant in the final multivariable model. This may be a result of the small number of observations, or a confounding between routine immunisation and tOPV campaigns, which are used to fill the immunity gaps. Finally, we also found that VDPV2 emergences were more likely to establish a circulating lineage and thus be responsible for more than one AFP case when they emerged in districts with low serotype-2 population immunity. Interestingly, the VDPV2 emergences that established a circulating lineage (7/29) occurred in districts with estimated serotype-2 population immunity <55%.

The statistical analyses of data on VDPV2 emergence in Nigeria are consistent with our transmission model results in suggesting that tOPV SIAs can in some settings increase the risk of VDPV emergence. Past experience has also highlighted how limited use of OPV (Sabin or other attenuated strains) either during small clinical trials (e.g. Poland [31,32]) or vaccination programmes (e.g. Byelorussia, former USSR [33]) can lead to widespread circulation of VDPV and outbreaks of poliomyelitis [31–33]. In Nigeria, the association that we found could be explained either by an insufficient number of campaigns, low coverage of those campaigns (settings with a higher number of campaigns may have poorer campaign coverage) or the existence of persistently “missed” populations, leading to insufficient levels of population immunity to avoid seeding new VDPV. Introduced in 2009 to monitor the quality of SIAs, lot quality assurance sampling (LQAS) showed that SIA coverage in 65% of districts in Nigeria did not reach 60% by the end of 2009 [34], suggesting that possibly only intermediate levels of SIA coverage were reached during the first half of the study period. Promisingly, significant improvements in SIA coverage have been reported since [34].

There are several limitations to our analyses. Firstly, the results of the case-control analyses were limited by the small number of emergence events in Nigeria, resulting in wide confidence intervals for some odds ratios. However, we chose Nigeria because it has experienced the greatest number of recorded VDPV2 emergences, with each VDPV2 undergoing detailed genetic sequencing and molecular epidemiological analysis [23]. Secondly, we were only able to examine risk factors associated with the first reported case of poliomyelitis caused by a VDPV2, rather than the initial emergence of the VDPV2. These VDPV2 isolates had between 6 and 17 nucleotide substitutions in the VP1 coding region, corresponding to an estimated average time of circulation since the initiating tOPV dose of around 9 months [23], thus leaving the possibility that the district where the first AFP case associated to a given emergence was detected did not correspond to the district where the initiating tOPV dose was administered. Thirdly, although our simple mathematical model is mechanistic–describing OPV transmission and VDPV emergence–it does not attempt to capture the detailed genetic changes that result in reversion of Sabin poliovirus to a VDPV with transmissibility and virulence equivalent to wild-type virus [8]. Attenuating mutations in the Sabin polioviruses have been identified, but the process of reversion and the significance of genetic changes for poliovirus transmission are not well understood [3]. Instead, we chose to capture the process of reversion by a simple probabilistic process and derive results that are likely to be robust to the details of genetic reversion. Fourth, although we considered models of immunisation that included persistently “missed” populations, we did not explicitly consider geographic heterogeneity in coverage and risk. Areas with poor routine immunisation coverage are also often challenging places to deliver vaccine during mass campaigns. These heterogeneities in coverage are therefore likely to increase the risk of VDPV2 emergence associated with tOPV SIAs, and it may be advisable to increase the number of campaigns in areas with heterogeneous coverage to account for this risk, as has been necessary during the eradication of wild-type polioviruses. Finally, other variables that could play a role in VDPV emergence such as hygiene behaviour, sanitation or the prevalence of other enterovirus serotypes (which could act as partners for recombination with Sabin viruses) were not included in the analysis of data from Nigeria, because they were not available at the district level.

Our transmission models do not account for the introduction of 1 or more doses of IPV into routine immunisation, which is a pre-requisite for OPV2 withdrawal [1]. Most countries currently using OPV only will introduce a single IPV dose at 14 or 16 weeks. This is likely to protect about 50% of vaccine recipients against poliomyelitis [35]. However, it is unclear what impact this vaccine will have on poliovirus transmission, which may well be limited as a result of the poor mucosal protection induced by this vaccine [36]. In particular, children born post OPV2 cessation (thus non-exposed to live serotype-2 virus) may not benefit from the boost in mucosal immunity induced by IPV [37,38]. Therefore, the impact of IPV on VDPV2 emergence and transmission may be limited [39], leaving populations at risk of silent transmission of poliovirus. Environmental surveillance will play a major role during the polio endgame, in particular to detect highly divergent Sabin-2 viruses that could be silently circulating and help to determine the extent of possible SIAs with monovalent OPV2 to control the spread of those and avoid new VDPV2 outbreaks.

Taken together, our results have important implications for efforts to prevent the emergence and subsequent spread of VDPVs during the polio endgame. First, they highlight the importance of enhancing routine immunisation coverage, which is already one of the main objectives of the Polio Eradication & Endgame Strategic Plan 2013–2018 [1]. Second, they may help to define a strategy for the use of tOPV in SIAs preceding the withdrawal of serotype 2 OPV that minimises the risk of VDPV2 post OPV2 withdrawal. In settings where population immunity is low and cVDPV2 currently absent, our findings and past experience suggest that 1 or 2 tOPV SIAs could increase the (small) risk of VDPV2 emergence compared to doing nothing. This risk is enhanced where tOPV immunogenicity is low, SIA coverage poor or there is a persistently “missed” group. Therefore, if tOPV SIAs are implemented preceding OPV2 withdrawal then they should be of sufficient number and high coverage to achieve high serotype-2 population immunity. In the context of limited resources, these SIAs should be targeted to countries considered at high risk of VDPV2 emergence according to the WHO risk assessment system. Finally, given the recently demonstrated advantage of IPV compared with OPV in terms of the boost to humoral and intestinal immunity in previously OPV-immunised children [37,38], IPV SIAs in high-risk areas may also be considered as part of the strategy to minimise the risk of VDPV2 emergence during OPV2 withdrawal.

## Supporting Information

S1 Text Supplementary Information. (PDF)
