# Supplementary material for: Preventing Vaccine-Derived Poliovirus Emergence during the Polio Endgame
Source: PLoS Pathog. 2016 Jul 6;12(7):e1005728. doi: 10.1371/journal.ppat.1005728 (PMC4934862; doi:10.1371/journal.ppat.1005728)
Supplement: S1 Text — (PDF) [file ppat.1005728.s001.pdf]

S1 Text – Supplementary Information

Preventing vaccine-derived poliovirus emergence during the polio endgame

Margarita Pons-Salort, Cara C. Burns, Hil Lyons, Isobel M. Blake, Hamid Jafari, M. Steven Oberste, Olen M. Kew, Nicholas C. Grassly

Contents

A Supplementary methods for the mathematical models **2**
- A.1 Analytical model . . . 2
  - A.1.1 Most Sabin secondary transmission from one SIA finishes before the next SIA . 2
  - A.1.2 Modelling details for the two types of SIA coverage: random and fixed . . . 2
- A.2 Reformulation of the analytical model . . . 3
  - A.2.1 Reformulation of the model by introducing the "effective vaccine coverage" . . 3
  - A.2.2 The two formulations of the analytical model are equivalent . . . 4
  - A.2.3 The minimal v or n that bring the probability of VDPV outbreak to zero . . . 5
- A.3 Stochastic compartmental model . . . 6

B Supplementary data for risk factors for VDPV2 emergence and spread in Nigeria (2004–2014) **8**

C Spatio-temporal modelling of serotype-2 population immunity **9**
- C.1 Methods . . . 9
- C.2 Results . . . 10

D Spatial modelling of DHS data through the SPDE approach **10**
- D.1 Methods . . . 10
  - D.1.1 General overview . . . 10
  - D.1.2 A hierarchical spatial model for DTP3 coverage . . . 11
  - D.1.3 A hierarchical spatial model for the mean number of household members . . . 11
  - D.1.4 Validation and prediction . . . 12
- D.2 Results . . . 12
  - D.2.1 Data, clusters and mesh . . . 12
  - D.2.2 Model parameter estimates . . . 12
  - D.2.3 Validation results . . . 12
  - D.2.4 Predictions . . . 13

E Supplementary Figures and Tables **14**

A Supplementary methods for the mathematical models

A.1 Analytical model

A.1.1 Most Sabin secondary transmission from one SIA finishes before the next SIA

It is reasonable to assume that all secondary transmission of Sabin poliovirus from one SIA has finished before the next SIA if $R_{0S} < 1$, because the majority of secondary transmission will take place in under 4 weeks. This can be shown by considering a branching process for OPV transmission: the cumulative distribution function for the number of generations g in an outbreak is given for a model with a geometric offspring distribution (which includes the simple SIR model) by Farrington & Grant, 1999 [1] as

$$P(G \leq g) = \frac{1 - R^{g+1}}{1 - R^{g+2}}$$

where $R < 1$ is the reproduction number. If we assume the generation time (interval between becoming infected and infecting others) for Sabin poliovirus transmission is approximately one week, consistent with estimates for wild-type poliovirus [2, 3], then 87% of transmission will have finished after 4 weeks for $R_{0S} = 0.9$. For lower values of R_{0S} transmission will be over faster, e.g. 98% by 4 weeks for $R_{0S} = 0.5$.

A.1.2 Modelling details for the two types of SIA coverage: random and fixed

After the first SIA, subsequent SIAs are assumed to reach the same proportion v of the population (i.e. same coverage), which may either consist of randomly chosen individuals at each round (random coverage), or repeatedly reach the same fixed group of individuals (fixed coverage), meaning there is a persistently "missed" group. Each of the subsequent n SIAs after the first one results in a proportion $i_2, i_3, ..., i_n$ of individuals shedding Sabin poliovirus. Similarly, we denote the proportion of susceptible following each SIA by $s_2, s_3, ..., s_n$.

In the case of random coverage,

$$i_j = y_1^j + y_2^j \qquad j = 2, ..., n$$

where y_1^j is the proportion shedding as a result of direct administration of OPV during the j-th SIA and y_2^j is the proportion shedding as a result of secondary spread of OPV from that SIA. Similar to the expressions for one SIA, those proportions are given by

$$y_1^j = s_{j-1} v w \tag{1}$$

$$y_2^j = (s_{j-1} - y_1^j) \left[1 - \exp(-R_{0S}(y_1^j + y_2^j))\right] \tag{2}$$

where v is the coverage of the campaign and w is vaccine "take". The proportion of susceptible following the j-th SIA is

$$s_j = s_{j-1} - i_j \qquad j = 2, ..., n.$$

In the case of fixed coverage, if we assume that secondary spread of OPV does reach the persistently missed group, then it becomes necessary to keep track separately of the fraction of the persistently missed group and of the vaccinated group that are susceptible to infection. In this model,

$$i_j = y_1^j + y_2^j = y_1^j + y_{2,V}^j + y_{2,M}^j$$

where $y_{2,V}^j$ and $y_{2,M}^j$ are the proportion of children who shed after secondary OPV spread following the j-th SIA in the vaccinated and missed groups respectively. The proportion shedding as a result of direct administration of OPV during the j-th SIA is $y_1^j = s_{j-1,V} v w$, where $s_{j-1,V}$ is the fraction of the vaccinated group that is susceptible to infection immediately before the j-th SIA commences. The proportion shedding as a result of secondary spread of OPV is given by

$$y_2^j = (s_{j-1,V} - y_1^j + s_{j-1,M}) \left[1 - \exp(-R_{0S}(y_1^j + y_2^j))\right]$$

where $s_{j-1,M}$ is the fraction of the persistently missed group susceptible to infection before the j-th SIA. A proportion p_V^j of y_2^j will belong to the vaccinated group, whereas a proportion $p_M^j = 1 - p_V^j$ will belong to the missed group, and those proportions will depend on the relative size of the susceptible in the two groups before the j-th SIA:

$$p_V^j = \frac{s_{j-1,V} - y_1^j}{s_{j-1,V} - y_1^j + s_{j-1,M}}$$

$$p_M^j = \frac{s_{j-1,M}}{s_{j-1,V} - y_1^j + s_{j-1,M}}.$$

Using p_V^j and p_M^j, the final proportions of individuals shedding as a result of secondary spread are given by

$$y_{2,V}^j = p_V^j y_2^j \qquad \text{and} \qquad y_{2,M}^j = p_M^j y_2^j$$

and the proportions of susceptible are finally updated as follows:

$$s_j = s_{j-1} - i_j$$

$$s_{j,V} = s_{j-1,V} - y_1^j - y_{2,V}^j$$

$$s_{j,M} = s_{j-1,M} - y_{2,M}^j.$$

Figure A illustrates the assumptions of this model.

A.2 Reformulation of the analytical model

The analytical model described in the main text and above (Section A.1) can be reformulated by changing the order in which OPV administration and secondary spread of OPV are assumed to occur. Instead of modeling the effect of n SIAs sequentially one by one (i.e. one SIA takes place, followed by the secondary spread of Sabin virus due to that SIA, then updating the proportion susceptible, and starting again the process as many times as the number of SIAs), we can model the same scenario as a single SIA with the proportion of individuals effectively immunised as a result of direct administration of OPV defined as a function of v, w and n, that will differ depending on whether we assume random or fixed coverage. To do this, we introduce the concept of "effective vaccine coverage", which is the proportion of the population that is effectively immunised as a result of direct administration of OPV after the n SIAs assuming no secondary spread of OPV from those SIAs. In other words, it is the proportion of the population for whom at least one dose of OPV has taken after the n campaigns, when we do not consider secondary spread of OPV between campaigns. Hereafter, we call the formulation of the analytical model presented in the main text as the "sequential" version, and the new formulation described below (in Section A.2.1) as the "simultaneous" version. We then show in Section A.2.2 that the two formulations of the analytical model are equivalent in the sense that the proportion of the population that has been infected with Sabin virus at the end of the n SIAs is the same.

A.2.1 Reformulation of the model by introducing the "effective vaccine coverage"

Given the number of SIAs n, the coverage of those SIAs v and the probability of vaccine take w, the "effective vaccine coverage" for the random and fixed coverage hypotheses, denoted by e_r and e_f respectively, can be written:

$$e_r = g_r w = 1 - (1 - vw)^n \qquad (3)$$

$$e_f = g_f w = v(1 - (1 - w)^n) \qquad (4)$$

As previously, we denote by s_0 the proportion of children susceptible to poliovirus before the SIAs commence. In this new formulation of the model, the proportion of children shedding vaccine virus after all the n SIAs is

$$\bar{i} = \bar{y_1} + \bar{y_2}$$

where $\bar{y_1}$ is the proportion shedding as a result of direct administration of OPV during the n SIAs, and $\bar{y_2}$ is the proportion shedding as a result of secondary spread of OPV. Note that with this new formulation, we have to compute $\bar{i}$ only once, whereas with the previous formulation, we computed i_j once for each SIA. Adapting the equations (1) and (2) for y_1^j and y_2^j, we can obtain the expressions for $\bar{y_1}$ and $\bar{y_2}$ as follows. In the case of random coverage,

$$\bar{y_1} = s_0 g_r w = s_0(1 - (1 - vw)^n) \tag{5}$$

whereas in the case of fixed coverage,

$$\bar{y_1} = s_0 g_f w = s_0 v(1 - (1 - w)^n). \tag{6}$$

The proportion shedding as a result of secondary spread of OPV is in both cases written as a function of $\bar{y_1}$ as

$$\bar{y_2} = (s_0 - \bar{y_1}) \left[1 - \exp(-R_{0S}(\bar{y_1} + \bar{y_2}))\right]. \tag{7}$$

Finally, the proportion susceptible after n SIAs, s_n, is given by:

$$s_n = s_0 - \bar{y_1} - \bar{y_2}. \tag{8}$$

This reformulation of the analytical model, although it may not seem natural, has several advantages and allows us to better study the behaviour of the system. In particular, using this new version of the model we can find an expression for the minimal SIA vaccine coverage or the minimal number of campaigns to have zero probability of a VDPV outbreak (Figure C and Section A.2.3).

A.2.2 The two formulations of the analytical model are equivalent

In this section, we show that the two formulations of the analytical model are equivalent for our purposes for $n = 2$. Using the same reasoning, the equivalence can be generalised for any $n \in \mathbb{N}^+$.

To prove that the two formulations ("sequential" and "simultaneous" versions) are equivalent, we need to show that the proportion of individuals who have been infected with Sabin after the n SIAs is the same with either version of the model. That is, using the notation introduced in Section A.1.2 and Section A.2.1, we want to see that the following equality is true:

$$\sum_{k=1}^{n} i_k = \bar{i}.$$

For $n = 2$, we thus need to show that $i_1 + i_2 = \bar{i}$, which is the same as:

$$y_1^1 + y_2^1 + y_1^2 + y_2^2 = \bar{y_1} + \bar{y_2}. \tag{9}$$

Note that the total proportion of individuals who have been infected with Sabin virus is the same in the two versions, but this is not true for the subproportions of primary infected with OPV as a result of direct administration of the vaccine (i.e. $y_1^1 + y_1^2 \neq \bar{y_1}$) or infected with OPV as a result of secondary spread (i.e. $y_2^1 + y_2^2 \neq \bar{y_2}$).

Without loss of generality, we can assume that $s_0 = 1$. Using the definitions of y_1^j ($j = 1, 2$) and $\bar{y_1}$ in the corresponding formulations of the model, we have:

$$y_1^1 = vw \tag{10}$$
$$y_1^2 = (1 - vw - y_2^1)vw \tag{11}$$
$$\bar{y_1} = 1 - (1 - vw)^2. \tag{12}$$

Replacing y_1^1, y_1^2 and $\bar{y_1}$ in (9) by their expressions ((10)–(12)), we need to show:

$$(1 - vw)y_2^1 + y_2^2 = \bar{y_2}. \tag{13}$$

Let's prove (13). We have given the definitions of y_1^1 and y_1^2 in (10) and (11) respectively. The definitions of y_2^1 and y_2^2 are:

$$\begin{aligned}
y_2^1 &= (1-y_1^1)\left[1-\exp\left(-R_{0S}(y_1^1+y_2^1)\right)\right] && (14)\\
&= (1-vw)\left[1-\exp\left(-R_{0S}(vw+y_2^1)\right)\right] && (15)\\
y_2^2 &= (1-y_1^1-y_2^1-y_1^2)\left[1-\exp\left(-R_{0S}(y_1^2+y_2^2)\right)\right] && (16)\\
&= (1-vw-y_2^1-vw(1-vw-y_2^1))\left[1-\exp\left(-R_{0S}(vw(1-vw-y_2^1)+y_2^2)\right)\right] && (17)\\
&= (1-vw)(1-vw-y_2^1)\left[1-\exp\left(-R_{0S}(vw(1-vw-y_2^1)+y_2^2)\right)\right] && (18)
\end{aligned}$$

Now, using (15) we obtain:

$$1-vw-y_2^1 = (1-vw)\exp\left(-R_{0S}(vw+y_2^1)\right) \tag{19}$$

and we can replace $(1-vw-y_2^1)$ in (18) by the expression in (19), thus giving:

$$y_2^2 = (1-vw)^2\exp\left(-R_{0S}(vw+y_2^1)\right)\left[1-\exp\left(-R_{0S}(vw(1-vw-y_2^1)+y_2^2)\right)\right]. \tag{20}$$

Using the expressions for y_2^1 and y_2^2 in (15) and (20) respectively, we can do the following calculus:

$$\begin{aligned}
(1-vw)y_2^1+y_2^2 &= (1-vw)^2\left[1-\exp\left(-R_{0S}(vw+y_2^1)\right)\right]+ && (21)\\
&+ (1-vw)^2\exp\left(-R_{0S}(vw+y_2^1)\right)\left[1-\exp\left(-R_{0S}(vw(1-vw-y_2^1)+y_2^2)\right)\right] && (22)\\
&= (1-vw)^2\left[1-\exp\left(-R_{0S}(vw+y_2^1)\right)\exp\left(-R_{0S}(vw(1-vw-y_2^1)+y_2^2)\right)\right] && (23)\\
&= (1-vw)^2\left[1-\exp\left(-R_{0S}(vw+y_2^1+vw(1-vw-y_2^1)+y_2^2)\right)\right] && (24)\\
&= (1-vw)^2\left[1-\exp\left(-R_{0S}(\underbrace{(1-(1-vw)^2)}_{\bar{y_1}}+\underbrace{(1-vw)y_2^1+y_2^2}_{\bar{y_2}})\right)\right]. && (25)
\end{aligned}$$

As the equation

$$\bar{y_2} = (1-vw)^2\left[1-\exp\left(-R_{0S}((1-(1-vw)^2)+\bar{y_2})\right)\right] \tag{26}$$

defines $\bar{y_2}$, we have proven that $(1-vw)y_2^1+y_2^2=\bar{y_2}$.

A.2.3 The minimal v or n that bring the probability of VDPV outbreak to zero

Using the simultaneous version of the analytical model we can find expressions for the minimal value of SIA vaccine coverage or the minimal number of campaigns that bring the probability of VDPV outbreak to zero.

The probability of VDPV outbreak is zero when

$$q = 1-\frac{1}{R_{0V}s_n} = 0 \iff s_n = \frac{1}{R_{0V}}$$

where s_n is the proportion susceptible after the n SIAs. Using the expression for s_n in the simultaneous version of the model, we can write:

$$q = 1-\frac{1}{R_{0V}s_n} = 0 \iff s_n = s_0-\bar{y_1}-\bar{y_2} = \frac{1}{R_{0V}}. \tag{27}$$

Using equation (27) and an approximation for $\bar{y_2}$ we can find an explicit expression to have zero probability of outbreak as a function $\bar{y_1}$ (i.e. as a function of the proportion of the population who will receive OPV and for whom the vaccine will take after the n campaigns). Using this, we'll be able to determine the minimal vaccine coverage or the minimal number of SIAs required to bring the probability of VDPV outbreak to zero, either for the random or fixed coverage.

We have seen in equation (7) that the proportion shedding as a result of secondary spread of OPV satisfies the equation

$$\bar{y_2} = (s_0-\bar{y_1})\left[1-\exp(-R_{0S}(\bar{y_1}+\bar{y_2}))\right].$$

A closed-form for $\bar{y_2}$ can be obtained by approximating $\exp(-R_{0S}\bar{y_2})$ by its first order Taylor expansion

$$\bar{y_2} \approx \frac{(s_0 - \bar{y_1})(1 - \exp(-R_{0S}\bar{y_1}))}{1 - (s_0 - \bar{y_1})R_{0S}\exp(-R_{0S}\bar{y_1})}. \tag{28}$$

Replacing $\bar{y_2}$ in equation (27) by its approximation in (28), we obtain

$$q = 0 \quad \Longleftrightarrow \quad s_0 - \bar{y_1} - \frac{(s_0 - \bar{y_1})(1 - \exp(-R_{0S}\bar{y_1}))}{1 - (s_0 - \bar{y_1})R_{0S}\exp(-R_{0S}\bar{y_1})} = \frac{1}{R_{0V}} \tag{29}$$

and rearranging it, we can find the following expression for zero probability of outbreak as a function of $\bar{y_1}$

$$q = 0 \quad \Longleftrightarrow \quad f(\bar{y_1}) = (s_0 - \bar{y_1})\exp(-R_{0S}\bar{y_1})\left[-(s_0 - \bar{y_1})R_{0S}R_{0V} + R_{0S} + R_{0V}\right] - 1 = 0. \tag{30}$$

If we denote by $\bar{y_1}^*$ the value of $\bar{y_1}$ such that $f(\bar{y_1}) = 0$, using the definition of $\bar{y_1}$ through the "effective vaccine coverage" in (5) and (6), we can find for a fixed number of campaigns n, the minimal value of the vaccine coverage to have zero probability of VDPV outbreak for the random and fixed coverage hypotheses, v^r_{min} and v^f_{min}, respectively:

$$v^r_{min} = \frac{1 - (1 - \frac{\bar{y_1}^*}{s_0})^{1/n}}{w} \quad \text{and} \quad v^f_{min} = \frac{\bar{y_1}^*}{s_0(1 - (1 - w)^n)}. \tag{31}$$

Inversely, for a fixed value of vaccine coverage v, we can find the minimal number of campaigns to have zero probability of VDPV outbreak for the random and fixed coverage hypotheses, n^r_{min} and n^f_{min}, respectively:

$$n^r_{min} = \left\lceil \frac{\ln(1 - \frac{\bar{y_1}^*}{s_0})}{\ln(1 - vw)} \right\rceil \quad \text{and} \quad n^f_{min} = \left\lceil \frac{\ln(1 - \frac{\bar{y_1}^*}{v s_0})}{\ln(1 - w)} \right\rceil.$$

Using the expressions in (31), we can study how the minimum value of SIA vaccine coverage required to have zero probability of VDPV outbreak decreases for an increasing number of SIAs:

$$\lim_{n\to\infty} v^{r,n}_{min} = \lim_{n\to\infty} \frac{1 - (1 - \frac{\bar{y_1}^*}{s_0})^{1/n}}{w} = 0$$

$$\lim_{n\to\infty} v^{f,n}_{min} = \lim_{n\to\infty} \frac{\bar{y_1}^*}{s_0(1 - (1 - w)^n)} = \frac{\bar{y_1}^*}{s_0}.$$

It is interesting to note that in the case of random coverage, for an increasing number of campaigns, the minimal SIA coverage to have zero probability of VDPV outbreak tends to 0. However, in the case of fixed coverage, this value tends to $\bar{y_1}^*/s_0$, meaning that after a certain number of campaigns, further increasing those will not reduce the value of vaccine coverage for which the probability of a VDPV outbreak is zero, and therefore, for values of vaccine coverage between $(0, \bar{y_1}^*/s_0)$, the probability of VDPV outbreak will remain greater than zero, due to the presence of the persistently "missed" group.

Using the expressions in (30) and (31), we can easily explore how the minimum value of vaccine coverage to have zero probability of VDPV outbreak changes for different values of the Sabin virus reproduction number, R_{0S}, and the VDPV reproduction number, R_{0V} (Figure D and Figure E).

A.3 Stochastic compartmental model

If a fixed proportion of individuals v_{ri} is reached by routine immunisation at each scheduled dose, and three doses of OPV are given, then assuming that only a fraction w of those doses will "take", the proportion c of children who received the three doses and shed Sabin virus is given by $c = v_{ri}w(1 + (1 - w) + (1 - w)^2)$. We model secondary spread of OPV through an SIR model with demography where the proportion c of children vaccinated and shedding the virus enter the model

as infected with Sabin poliovirus, and the other children enter the model as susceptible. A small proportion of incident Sabin poliovirus infections are assumed to revert to VDPV, which may then spread in the population. We assume that this proportion is fixed and independent of whether infection was acquired directly through immunisation or through secondary spread of OPV.

The model is described by the following set of differential equations:

$$\begin{aligned}\frac{dx}{dt} &= (1-c)\mu - \beta_S xy - \beta_V xv - \mu x \\ \frac{dy}{dt} &= (1-\rho)c\mu + (1-\rho)\beta_S xy - \gamma y - \mu y \\ \frac{dz}{dt} &= \gamma y + \gamma v - \mu z \\ \frac{dv}{dt} &= \rho c\mu + \rho\beta_S xy + \beta_V xv - \gamma v - \mu v\end{aligned}$$

where x, y, z and v represent respectively, the proportion of individuals susceptible, infected with Sabin virus, immune, or infected with a VDPV. The parameter μ is the rate at which children enter and leave the system, and thus $1/\mu$ is the average age of the population under consideration (set to 4 years). The mean duration of infectiousness was $1/\gamma = 4$ weeks [4], assumed to be the same for Sabin virus and VDPVs. β_S and β_V determine the rate at which individuals infected with Sabin poliovirus or VDPVs, respectively, infect susceptible individuals in the population. The reproduction number of Sabin virus and VDPVs are given by $R_{0S} = \beta_S/(\gamma + \mu)$ and $R_{0V} = \beta_V/(\gamma + \mu)$. Finally, ρ is the probability that a Sabin infection reverts to a VDPV.

We denote by v_{sia} the proportion of children reached by each campaign. If we assume SIA random coverage (i.e the proportion v_{sia} of the population reached at each SIA consists of randomly chosen individuals at each round), at each supplementary campaign a proportion $(1-\rho)v_{sia}w$ of susceptible individuals becomes infected with Sabin virus, and a proportion $\rho v_{sia}w$ becomes infected with a VDPV. We further assume that SIA are implemented 4 weeks apart.

Because VDPVs are rare, we assume that no VDPVs circulate at $t = 0$, and we set the initial conditions at the equilibrium of the nested model without VDPVs (i.e. $v(0) = 0$ and $\rho = 0$):

$$(x(0), y(0), z(0)) = \left(\frac{1}{2}(1+\Delta), \frac{\mu}{2(\mu+\gamma)}(1-\Delta), \frac{\gamma}{2(\mu+\gamma)}(1-\Delta)\right)$$

where

$$\Delta = \frac{1}{R_{0S}} - \sqrt{1 + \frac{4c-2}{R_{0S}} + \frac{1}{R_{0S}^2}}.$$

We implemented a stochastic version of the model using the Gillespie's τ-leap algorithm with a time step of 0.1 week. Table A lists all the events describing the stochastic model for SIA random coverage.

To adapt the dynamic model to assume SIA fixed coverage (i.e. the SIAs repeatedly reach the same fixed group of individuals of size v_{sia}), we need to split the population into two groups: a "vaccinated" group that will represent a proportion v_{sia} of the population, and a "missed" group that will be the rest of the population, $1 - v_{sia}$. That is, for each variable x, y, v and z, we now have two versions: x_V, y_V, v_V and z_V, for the individuals in the "vaccinated" group, and x_M, y_M, v_M and z_M, for the individuals in the "missed" group. The dynamics of Sabin and VDPV transmission are the same. In this case, at each supplementary campaign, a proportion $(1-\rho)w$ of susceptible individuals in the "vaccinated" group becomes infected with Sabin virus, and a proportion ρw of those becomes infected with a VDPV. Note that we assume that routine immunisation coverage is independent of the SIA coverage. A list with all the events in the SIA fixed coverage model is provided in Table B.

B Supplementary data for risk factors for VDPV2 emergence and spread in Nigeria (2004–2014)

Independent VDPV2 emergences. Data on the state, district and 6-month period where the first case of poliomyelitis associated with each of the 29 independent VDPV2 emergences reported in Nigeria during 2004–2014 are given in Table C.

Serotype-2 population immunity. We estimated serotype-2 vaccine-induced population immunity by district and 6 months from vaccine efficacy [5] and coverage estimates based on AFP data for children in Nigeria <36 months old reported during 2004–2014 using previously published methods [6, 5] and a random-effects spatio-temporal model (see Section C for the details).

Routine immunisation coverage. Estimates of routine immunisation coverage were obtained using data from the 2003, 2008 and 2013 Demographic and Health Surveys (DHS) [7]. Because a large number of OPV doses in Nigeria are given through supplementary campaigns, and DHS do not distinguish between routine and supplementary vaccinations, we used data for diphtheria-tetanus-pertussis (DTP) vaccination instead of OPV, as the former is a better indicator of the children who attend health services for routine immunisation. The "children DHS surveys" provide individual and spatial data: for each child, the DTP doses received and the geographical coordinates (latitude and longitude) of the sample point (cluster) with an error of less than 2km are reported. A child was assumed fully vaccinated through routine immunisation if he/she had received the third dose of DTP (DTP3) by the age of 12 months or later. Those children for whom the age was not reported were excluded. For each cluster (i.e. sample point, which is likely to correspond to a village or a city), we calculated the proportion of fully vaccinated children and then used a spatial Stochastic Partial Differential Equation (SPDE) model [8] implemented with the R-INLA package [9, 10] to obtain predictions at unobserved locations over Nigeria conditional on the observed data. We then used administrative boundary data to extract mean summaries by district (see Section D for the details). We repeated the procedure for the three surveys (2003, 2008 and 2013), thus giving 3 estimates per district, and finally used linear interpolation to obtain estimates of routine immunisation coverage for every 6-month period.

Number of tOPV SIA campaigns in the past 6 months and time since the last tOPV SIA. For each district and 6-month period, the number of SIA campaigns using tOPV in the preceding 6 months were extracted from the SIA calendar maintained by WHO (Figure H). The number of months between the last SIA campaign using tOPV and the start of each 6-month period was also calculated.

Number of births. Estimates for the annual number of births per district were based on data for 2010 from WorldPop [11] and data on administrative boundaries in Nigeria. WorldPop provides estimates of live births per grid square in squares of approximately 100m side. Those estimates were aggregated per district using the borders given by the shapefile (Figure I). For each district, the estimate for 2010 was used over the entire period.

Population density. For each district, the population density was calculated using the data for total population size and land size reported in the Census of 2006 [12] (Figure J). For each district, the estimate for 2006 was used over the entire period.

Mean number of household members. Estimates for the mean number of household members in each district were obtained using data from the 2003, 2008 and 2013 DHS [7]. The "household DHS surveys" provide data for the number of household members and the geographical coordinates (latitude and longitude) of the sample point (cluster) with an error of less than 2km. We calculated the mean number of members per household in a cluster and then proceeded as for routine immunisation to get estimates per district per 6-month period. See Section D for the details.

C Spatio-temporal modelling of serotype-2 population immunity

C.1 Methods

We estimated serotype-2 population immunity for discrete 6-month periods for each district based on the number of doses of OPV reported by non-polio AFP cases <36 months of age in that district and the SIA calendar. We calculated estimates for each one year age group and took an average across these age groups (since each age group is approximately equal in size).

In brief, individual serotype-2 immunity is obtained using the number of tOPV doses received and a per-dose efficacy against serotype 2 poliomyelitis (previously estimated at 0.485 [5]), assuming an all-or-nothing protective response to vaccination where the per-dose probability of inducing an immune response is independent of the previous doses, proceeding as in previously published methods [6, 5]. Because the reported number of doses does not specify the vaccine type, we derived the number of tOPV doses that a non-polio AFP case had received by multiplying the number of reported doses by the proportion of SIAs with tOPV that the child was exposed to based on the SIA calendar.

The reported doses do not distinguish between routine and supplementary doses either. We thus used the estimates of routine immunisation coverage (obtained in Section D) to account for the doses received through routine immunisation. For each non-polio AFP case, we generated 1000 possible vaccination histories by defining a binary variable indicating whether the child was immunised through routine services, drawn from a Bernoulli distribution with probability of success given by the routine immunisation coverage in that district 6-month period. For each random draw, if a child was assumed to participate in routine immunisation, three of the total number of doses reported (or the maximum of doses reported, if <3 doses were reported) were assumed to be tOPV –corresponding to the routine immunisation doses– and the remainder were assumed to be received through SIAs. We therefore generated 1000 estimates of the probability of serotype-2 immunity per child, which were used to obtain 1000 estimates of immunity for each district 6-month period by averaging across all children and age group. We then used the median of those estimates in subsequent analyses.

We fitted a random-effects spatio-temporal model to the median estimates. This was important, because the number of non-polio AFP cases by district 6-month period is relatively small, sometimes zero, leading to districts 6-month period with no estimate or with estimates unusually low or high, extremely affected by the presence of outliers.

Let p_{ijt} be the median estimate of population immunity in a district i (in a state j) at a 6-month period t, and n_{ijt} the number of non-polio AFP cases used to estimate p_{ijt}. We defined the following random-effects model for p_{ijt}:

$$p_{ijt} \sim \text{Normal}\left(\eta_{ijt}, \frac{\sigma^2}{n_{ijt}}\right)$$

$$\text{logit}(\eta_{ijt}) = \alpha + \gamma_{it} + \delta_{jt}$$

where α is the intercept and γ_{it} and δ_{jt} are two space-time interactions, at two different space levels, the district, γ, and the state, δ. Having the state-level effect is important, because most of the SIA are organised at the state level. The district level accounts for differences in vaccine coverage within states. The interactions with time are necessary, because the number of tOPV SIA has highly changed over the study period (see Figure H) and because changes in coverage are also likely to have occurred. We assume that both interactions have a structured temporal effect and an unstructured spatial effect, and we assume an autoregressive time structure of second order. That is, we assume a second-order random walk across time for each area (district or state) independently of all other areas. The time unit is 6 months.

Let T be the total number of time periods, I the total number of districts and J the total number of states. The densities of the two random effects can be written as

$$\pi(\gamma|\sigma_\gamma^2) \propto \exp\left(-\frac{1}{2\sigma_\gamma^2}\sum_{t=3}^{T}\sum_{i=1}^{I}(\gamma_{i,t} - 2\gamma_{i,t-1} + \gamma_{i,t-2})^2\right)$$

and

$$\pi(\delta|\sigma_\delta^2) \propto \exp\left(-\frac{1}{2\sigma_\delta^2}\sum_{t=3}^{T}\sum_{j=1}^{J}(\delta_{j,t} - 2\delta_{j,t-1} + \delta_{j,t-2})^2\right)$$

respectively [13]. Other models have been used in the literature to smooth crude estimates of population immunity (e.g. [14]).

The model was fitted to the data using the Integrated Nested Laplace Approximation (INLA) approach [9] implemented in the R-INLA package [10]. The INLA algorithm, proposed by Rue et al. (2009), is a computationally efficient method for Bayesian inference with latent Gaussian models [9]. This approach provides accurate approximations of the posterior distributions in lower computational time than Markov Chain Monte Carlo methods.

C.2 Results

The intercept, α, and the precision for the Gaussian observations and the two space-time interactions ($1/\sigma^2$, $1/\sigma_\gamma^2$ and $1/\sigma_\delta^2$) were estimated (Table D). The estimated serotype-2 population immunity per district per 6-month period is illustrated in Figure G.

D Spatial modelling of DHS data through the SPDE approach

D.1 Methods

D.1.1 General overview

The goal was to obtain predictions of two variables (namely, DTP3 coverage and mean number of household members) at unobserved locations conditionally on the observed data to produce a continuous map of those variables in Nigeria. For this, we fitted a Bayesian hierarchical spatial model to data provided by three Demographic and Health Surveys (DHS): 2003, 2008 and 2013 [7]. The models were implemented through the Stochastic Partial Differential Equation (SPDE) approach and we used Integrated Nested Laplace Approximation (INLA) for inference. The ultimate goal was to use those maps to obtain an estimation of the two variables of interest per district, for the three years of the surveys.

The SPDE approach [8] is useful to implement spatial models for point-reference data. The basic idea of this approach is to represent a Gaussian field (GF) with continuous Matérn covariance function as a discretely indexed Gaussian Markov random field (GMRF), and then do the computations using the GMRF representation. The advantage of working with GMRFs is that, due to the Markovian property (i.e. the full conditional distribution of the GF in one point depends only on the neighbours of that point), they are defined by sparse precision matrices (i.e. the inverse matrix of the covariance matrix), which makes numerical methods computationally more efficient.

The Matérn covariance Σ between two points with locations s_i and $s_j \in \mathbb{R}^d$ is defined as

$$\Sigma_{ij} = \text{cov}(x(s_i), x(s_j)) = \frac{\sigma^2}{2^{\nu-1}\Gamma(\nu)}(\kappa||s_i - s_j||)^\nu K_\nu(\kappa||s_i - s_j||)$$

where $||\cdot||$ is the Euclidean distance in $\mathbb{R}^d$, σ^2 is the marginal variance, and K_ν denotes the modified Bessel function of second kind and order $\nu > 0$. The parameter ν is usually kept fixed and relates to the smoothness of the underlying process and the scaling parameter $\kappa > 0$ is related to the spatial range of the covariance function (i.e. the distance at which the spatial correlation becomes almost null). Based on this, the parameter range ρ has been empirically defined as $\rho = \sqrt{8\nu}/\kappa$, corresponding to distances at which correlation is near 0.13, for all $\nu > 1/2$ [15].

In the SPDE approach, the GMRF that best represents a given GF with Matérn covariance is constructed by using a certain SPDE that has such GF as a solution. The GMRF is formulated as a finite weighted sum of simple basis functions and Gaussian-distributed weights. For a triangulation

of the spatial domain and some chosen simple local basis functions (e.g. piecewise linear functions in each triangle), the joint distribution of the weights is chosen so that the finite element representation approximates the solutions to the SPDE. Lindgren et al. (2011) provided an explicit link between the parameters of the GF (dense) covariance function and the Gaussian weights of the (sparse) precision matrix of the GMRF. The values in the interior of the triangles are then determined by linear interpolation. We refer to Lindgren et al. (2011) for the details of the SPDE approach [8].

D.1.2 A hierarchical spatial model for DTP3 coverage

A Bayesian hierarchical spatial model for the outcome variable DTP3 coverage was implemented through the SPDE approach with R-INLA [10] and fitted to three datasets provided by the 2003, 2008 and 2013 DHS [7].

We assume that the proportion of DTP3 coverage is binomially distributed:

$$y_i \sim \text{Binomial}(n_i, p_i)$$

where y_i is the observed value of DTP3 coverage at the DHS cluster i. This data model describes how observations arise assuming a known latent field for the parameter p. We then model the probability p as a logit-transformed Gaussian process using a stochastic model that only includes an intercept α, and spatial structure (i.e. we do not include any covariates), resulting in the following latent Gaussian model:

$$\text{logit}(p_i) = \alpha + x_i$$

$$\mathbf{x} \sim \text{Normal}(0, \mathbf{\Sigma})$$

where $\mathbf{x}$ is a mean-zero Gaussian field with Matérn spatial covariance, defining the spatially structured random effect of the model. Finally, we used the default priors for the parameters implemented in R-INLA [10].

D.1.3 A hierarchical spatial model for the mean number of household members

A Bayesian hierarchical spatial model for the outcome variable mean number of household members was also implemented through the SPDE approach with R-INLA [10] and fitted to three datasets provided by the 2003, 2008 and 2013 DHS [7].

Because the mean number of household members is always positive, we assumed that it followed a Gamma distribution:

$$y_i \sim \Gamma(\mu_i, c\phi)$$

and we used the parameterisation of the Gamma distribution used by INLA:

$$\pi(y) = \frac{1}{\Gamma(c\phi)}\left(\frac{c\phi}{\mu}\right)^{c\phi} y^{c\phi-1}\exp(-c\phi\frac{y}{\mu})$$

where c is a fixed scaling parameter and ϕ a precision parameter (or $1/\phi$ a dispersion parameter). We model the mean μ as a log-transformed Gaussian process using a stochastic model that, as previously, only includes an intercept α, and spatial structure (i.e. we do not include any covariates), resulting in the following latent Gaussian model:

$$\log(\mu_i) = \alpha + x_i$$

$$\mathbf{x} \sim \text{Normal}(0, \mathbf{\Sigma})$$

where $\mathbf{x}$ is again a mean-zero Gaussian field with Matérn spatial covariance, defining the spatially structured random effect of the model. Again, we used the default priors for the parameters implemented in R-INLA [10].

D.1.4 Validation and prediction

In order to validate the models, we used out-of-sample predictions with 10 different samples. Each sample was constructed by randomly removing 5% of the observations from the dataset. We then fitted the model with the remaining observations and used the removed observations for validation purposes. For each model and each sample, we computed two predictive performance statistics: the mean squared error (MSE) and the Pearson correlation coefficient.

Using the estimates of the model parameters, we estimated the continuous field for the mean and the standard deviation of the variables of interest on a regular grid covering Nigeria.

Finally, the value of the variable of interest per district was taken as the mean of the values of the variable on the points of the grid inside the polygon defining the boundaries of that district.

D.2 Results

D.2.1 Data, clusters and mesh

The observations used to fit the models are the values of the two variables (DTP3 coverage and mean number of household members) observed in the clusters of the DHS. Figure K shows the location of the clusters and the value of the two variables reported in each cluster. The number of observations (i.e. clusters) significantly increased between the survey of 2003 and the survey of 2008 and remained nearly the same between 2008 and 2013 (Table E). There was a similar increase in the number of observations per cluster. For DTP3 coverage, the mean number of observations per cluster was 10.4 (interquartile range [IQR], 5.5–14.0) in 2003, 20.98 (IQR 13.00–28.00) in 2008, and 23.84 (IQR 15.00–32.00) in 2013, whereas for the mean number of household members, the mean number of observations per cluster was 19.96 (IQR 18.00–23.00) in 2003, 38.45 (IQR 37.00–41.00) in 2008 and 42.99 (IQR 42.00–45.00) in 2013.

We used the function implemented in R-INLA to construct the meshes to calculate the GMRFs in the SPDE approach. By default, this function constructs a constrained refined Delaunay triangulation. Because we used the location of the clusters as nodes of the mesh, the number of triangles of the mesh also increased between 2003 and 2008, and remained similar between 2008 and 2013 (Table E).

D.2.2 Model parameter estimates

Results for the parameter estimates are shown in Table F for the models of DTP3 coverage and Table G for the models of the mean number of household members.

For DTP3 coverage, the value of the intercept α increased over time, probably due to a general increase in vaccine coverage. The spatial correlation range ρ, diminished between the model for 2003 (149 km, 95% credible interval [CI] 101–213) and the model for 2008 (83 km, 95% CI 71–97), and remained very close for 2013 (84 km, 95% CI 72–97). This is probably related to the number of data points, that increased between 2003 and 2008, and then remained nearly the same.

For the mean number of household members, the intercept α slightly diminished between 2003 and 2008 and then remained similar for 2013. This is probably due to a general decrease in the number of household members. The spatial correlation range ρ decreased over time from 560 km (95% CI 289–1036) in 2003 to 241 km (95% CI 172–333) in 2013, but remained very high, suggesting that the data has a large spatial correlation. The same parameter was much lower for the models of DTP3 coverage, possibly indicating more spatial variability for this latter variable.

D.2.3 Validation results

Despite their simplicity (the models relied only on an intercept and spatial structure; no covariates were included), the six models showed a good predictive power, as indicated by relatively high values of the Pearson correlation coefficient (mean values > 0.55) and low values for the Mean Squared

Error, showing good agreement between the observed and predicted values based on the out-of-sample predictions (Figure N). Moreover, the prediction performance seemed to improve over time, with both statistics markedly improving when the models were fitted to the datasets of 2008 and 2013. This improvement is probably due to the increasing number of observations points (i.e. clusters) in the DHS (see Table E).

D.2.4 Predictions

The mean and standard deviation of the predicted spatial fields for DTP3 coverage and mean number of household members are shown respectively in Figure O and Figure P. Finally, the estimated mean value of the two variables per district are illustrated in Figure 4B of the main text and Figure Q respectivley.

E Supplementary Figures and Tables

Figure A: Illustration of the analytical model assumptions. This figure describes the transmission of Sabin virus following the implementation of one SIA with OPV in a population that is partially immune. The total population size is $N = 40$. (A) A proportion of the population s_0 is susceptible (gray) at the time that the SIA commences, and thus a proportion $1 - s_0$ is immune (blue). (B) In this example, we assume that the SIA reaches a proportion $v = 70\%$ of the population. (C) We assume that OPV does not replicate in immune individuals. However, susceptible individuals who receive a dose of OPV will shed Sabin virus if the vaccine takes. We fix the probability of vaccine take at $w = 55\%$. We denote by y_1^1 the proportion of the population that sheds Sabin virus due to direct administration of the vaccine (red). (D) Sabin-infected individuals may spread the virus to their susceptible contacts. The reproduction number of Sabin virus is assumed to be small, $R_{0S} < 1$, meaning that the proportion of individuals who will become infected due to secondary spread of OPV will also be small. The proportion of the population that becomes infected with Sabin due to a contact with a Sabin-infected individual (and not because of direct administration of OPV) is denoted by y_2^1 (orange). (E) We assume that all Sabin infections have the same, very small probability of reverting to a VDPV (green). All the individuals who have been infected by Sabin virus become immune, and the proportion who remains susceptible after the SIA is updated by removing those who have been infected. (F) The Sabin viruses that have reverted to VDPVs have a certain probability of spreading in the population that will depend on the proportion of individuals susceptible after the campaigns (in this example, s_1) and the transmission number of the VDPVs, $R_{0V} > 1$.

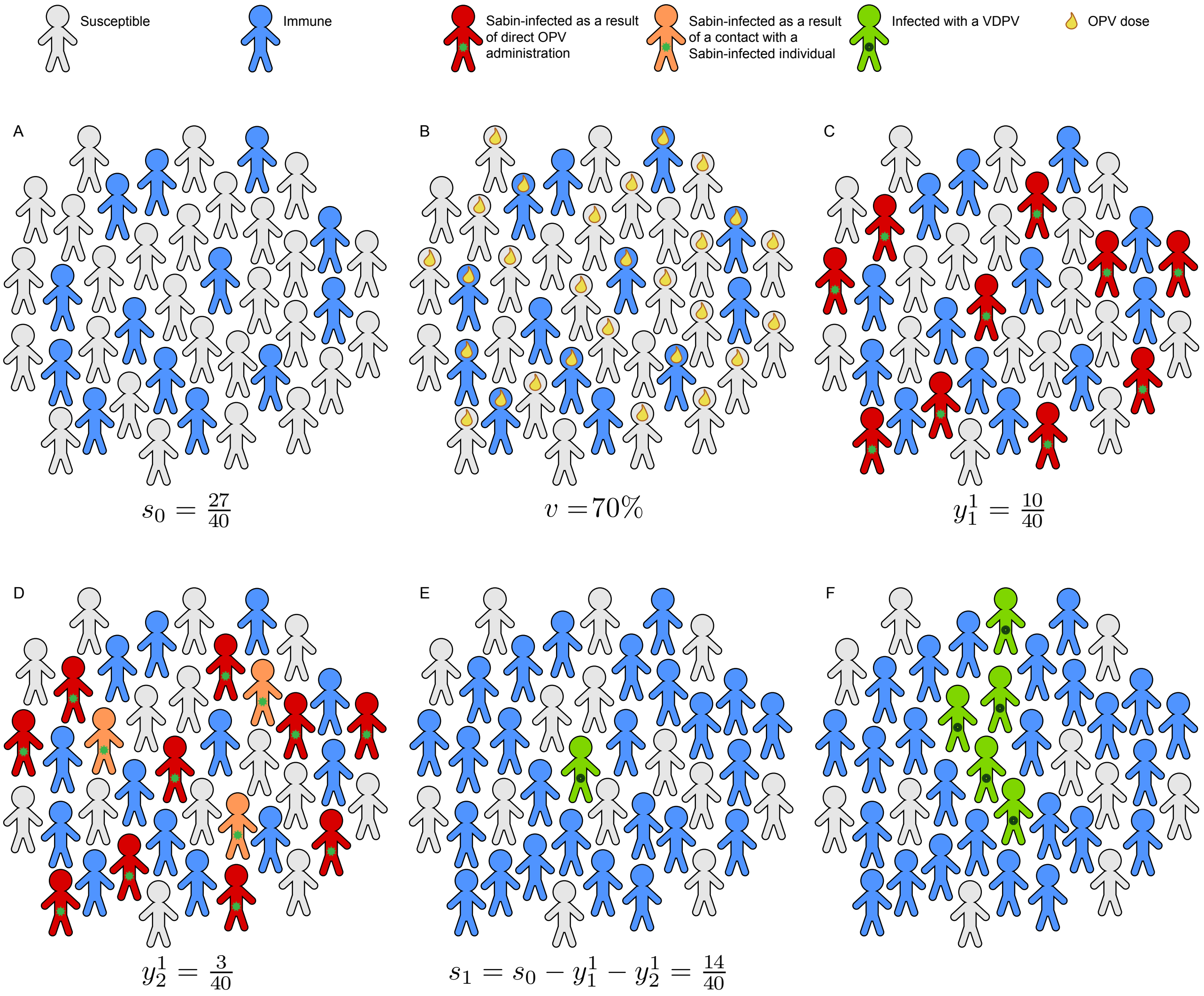

Figure B: Sensitivity analysis of the probability of a VDPV outbreak to the parameter σ (analytical model). The probability of a VDPV outbreak depends on the probability of Sabin reversion (ρ) and the population size (N), via $\sigma = \rho N$, implying that for any pair of values (ρ,N) that have the same product, the behaviour of the system is the same. In the main text, we have explored the probability of a VDPV outbreak for $\sigma = 5$. Here we show how the probability of a VDPV outbreak changes for smaller ($\sigma = 1$) and larger ($\sigma = 50$) values of σ. Probability of a VDPV outbreak for $\sigma = 1$, assuming random (A) and fixed (B) coverage, and for $\sigma = 50$, also assuming random (C) and fixed (D) coverage.

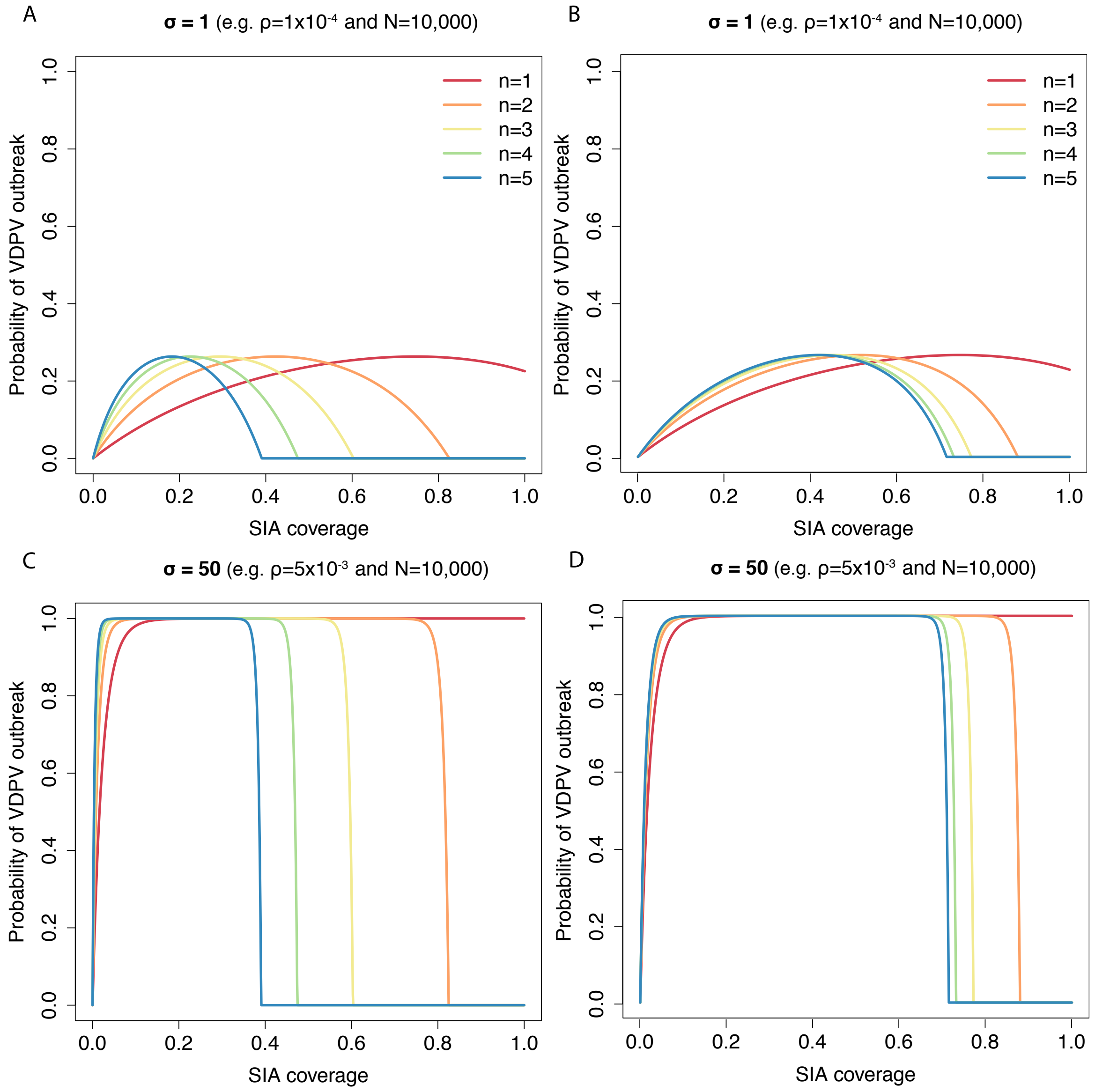

Figure C: Illustration of results that can be obtained with the "simultaneous" version of the analytical model. In Section A.2.3 we show how we can find an expression for the minimal value of SIA vaccine coverage or the minimum number of SIAs for which the probability of VDPV outbreak is zero (blue arrow).

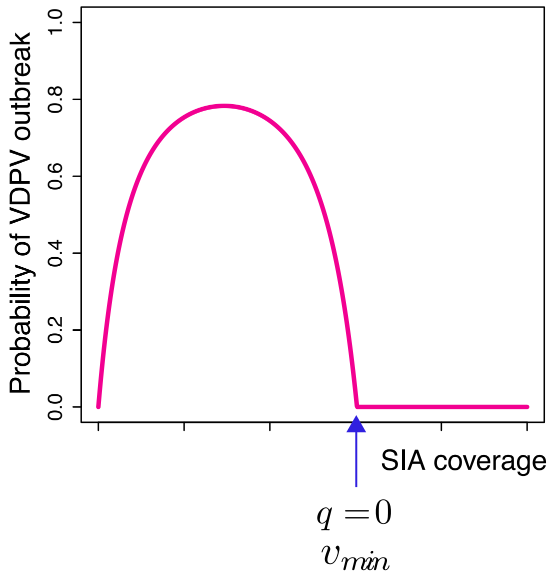

Figure D: Sensitivity analysis of the critical value of SIA vaccine coverage for $s_0 = 1$. Minimum value of SIA vaccine coverage to have zero probability of a VDPV outbreak for different values of the Sabin virus reproduction number, R_{0S} (x-axis), and different values of the VDPV reproduction number, R_{0V} (y-axis), under the hypotheses of random (left) and fixed (right) coverage and for 2, 3 and 4 rounds of SIA (from top to bottom), in a population completely susceptible (i.e. $s_0 = 1$). White areas indicate that the probability of a VDPV outbreak cannot be zero even when 100% of the population is vaccinated in each SIA.

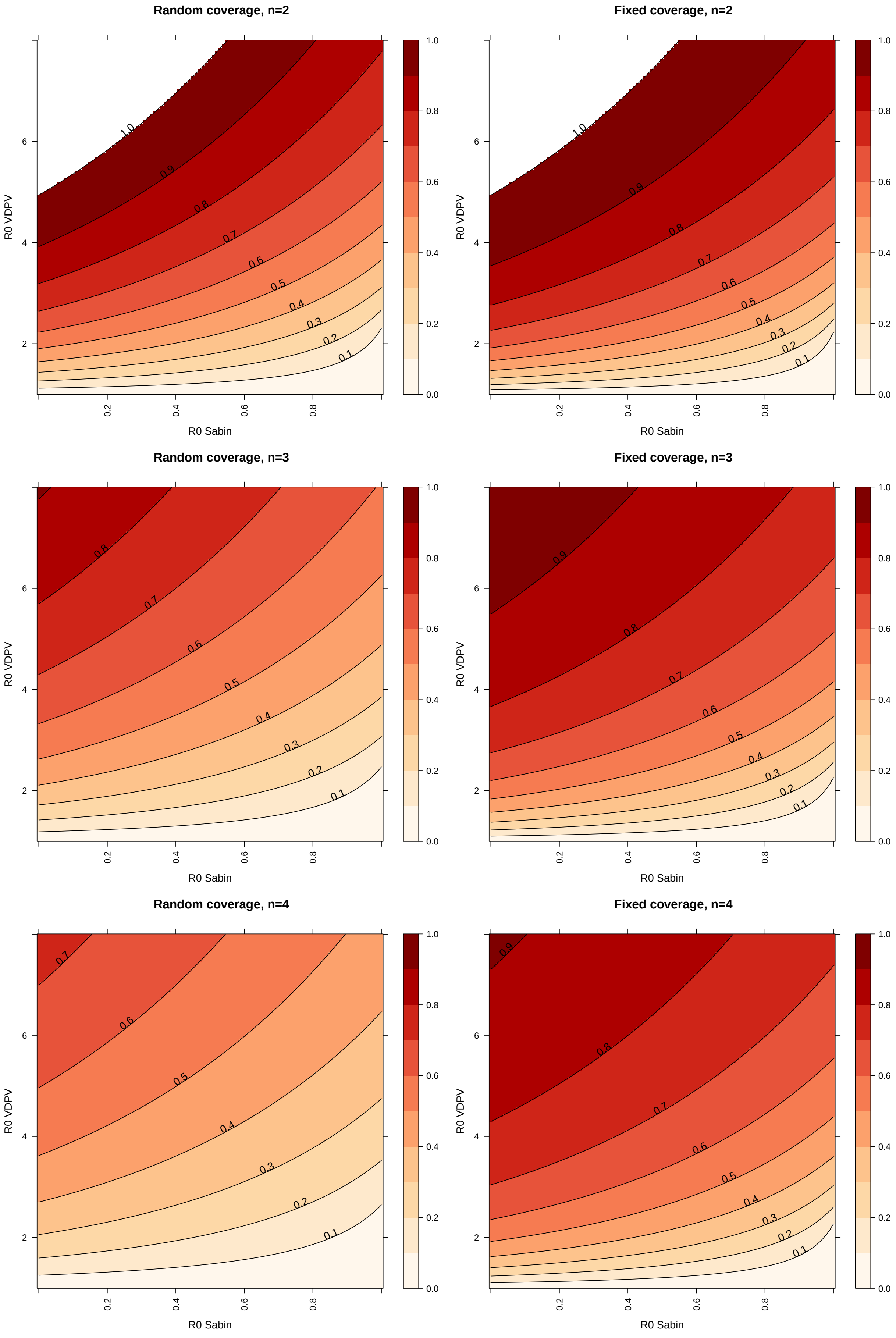

Figure E: Sensitivity analysis of the critical value of SIA vaccine coverage for $s_0 = 0.6$. Minimum value of SIA vaccine coverage to have zero probability of a VDPV outbreak for different values of the Sabin virus reproduction number, R_{0S} (x-axis), and different values of the VDPV reproduction number, R_{0V} (y-axis), under the hypotheses of random (left) and fixed (right) coverage and for 2, 3 and 4 rounds of SIA (from top to bottom), in a population where 60% of the children are susceptible to poliovirus (i.e. $s_0 = 0.6$).

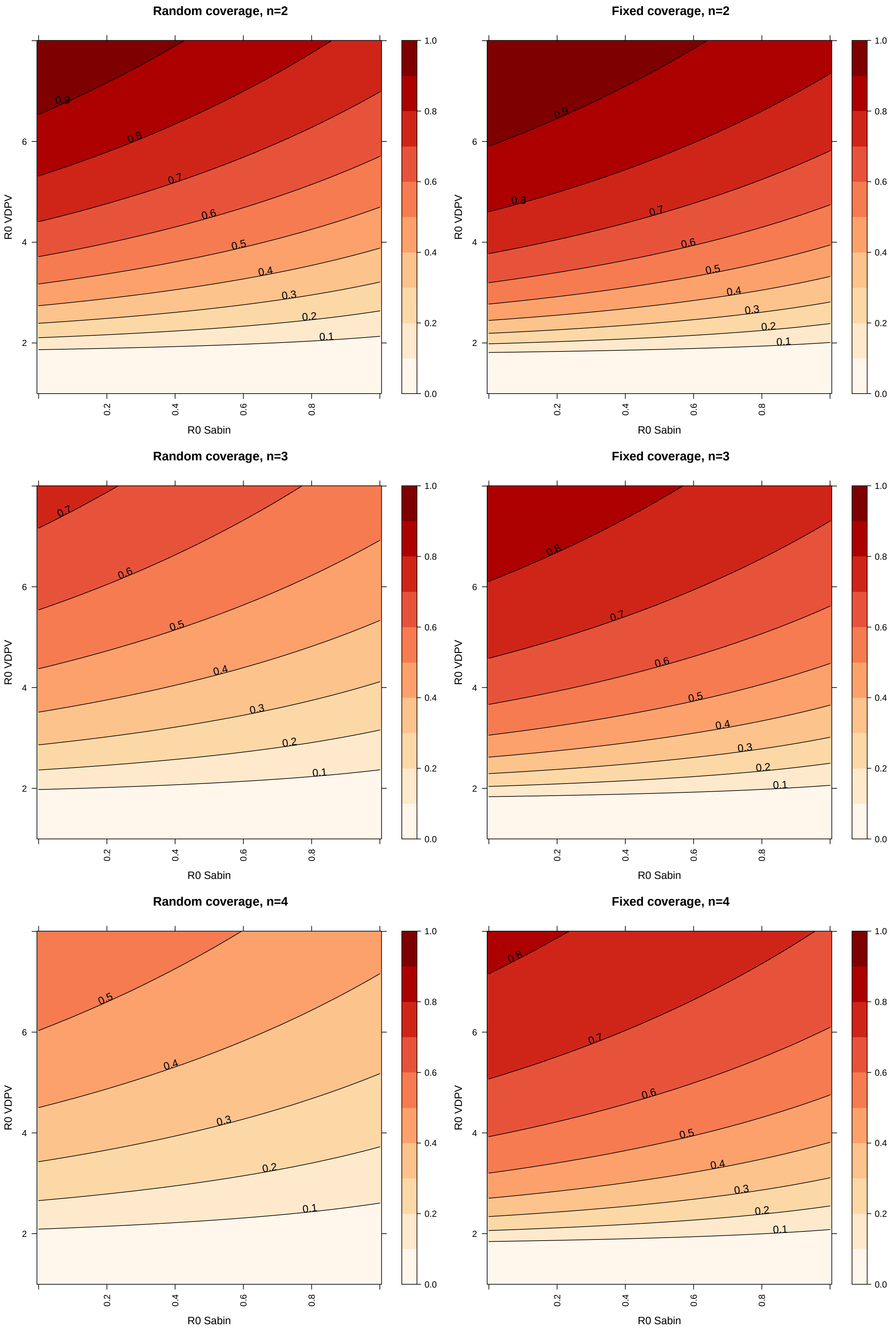

Figure F: Sensitivity analysis of the probability of a VDPV outbreak to the population size (N) for the stochastic transmission model. The probability of a VDPV outbreak is defined as the probability of >200 VDPV2 incident infections in the 6 months following OPV2 withdrawal. We show this probability for increasing population size: (A) 10 000, (B) 100 000, and (C) 1 000 000, while keeping the product of the probability of Sabin reversion (ρ) and the population size (N) constant.

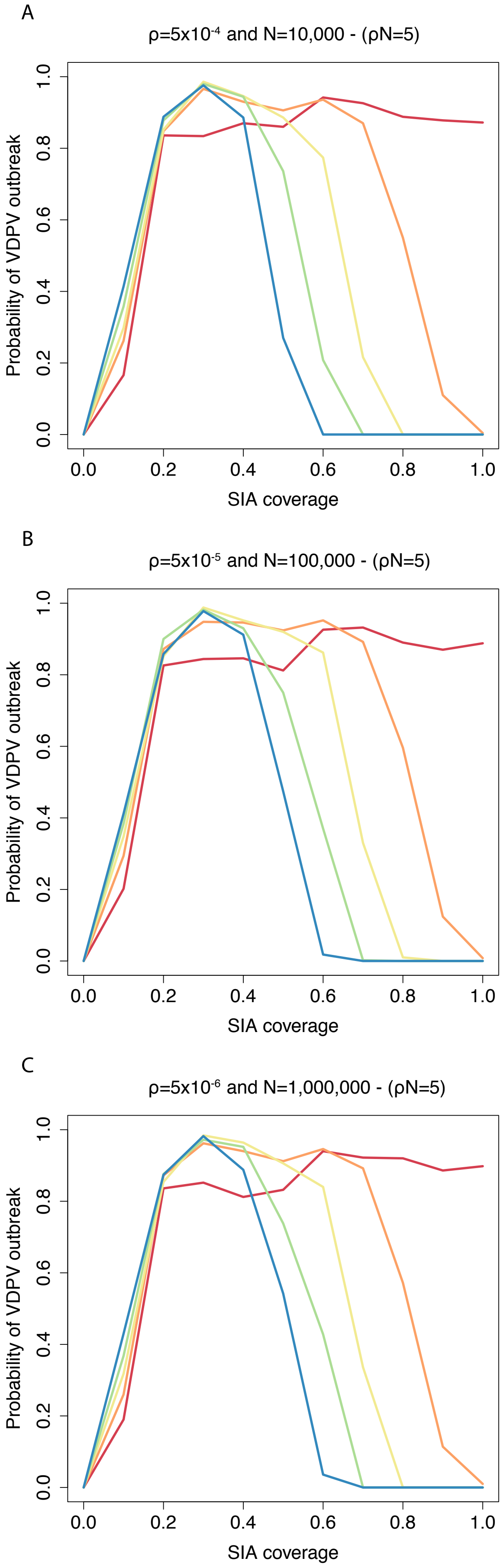

Figure G: Serotype-2 population immunity estimates per district and 6-month period. The publication of this map does not imply the expression of any opinion whatsoever on the part of WHO concerning the legal status of any territory, city or area or of its authorities, or concerning the delimitation of its frontiers or boundaries.

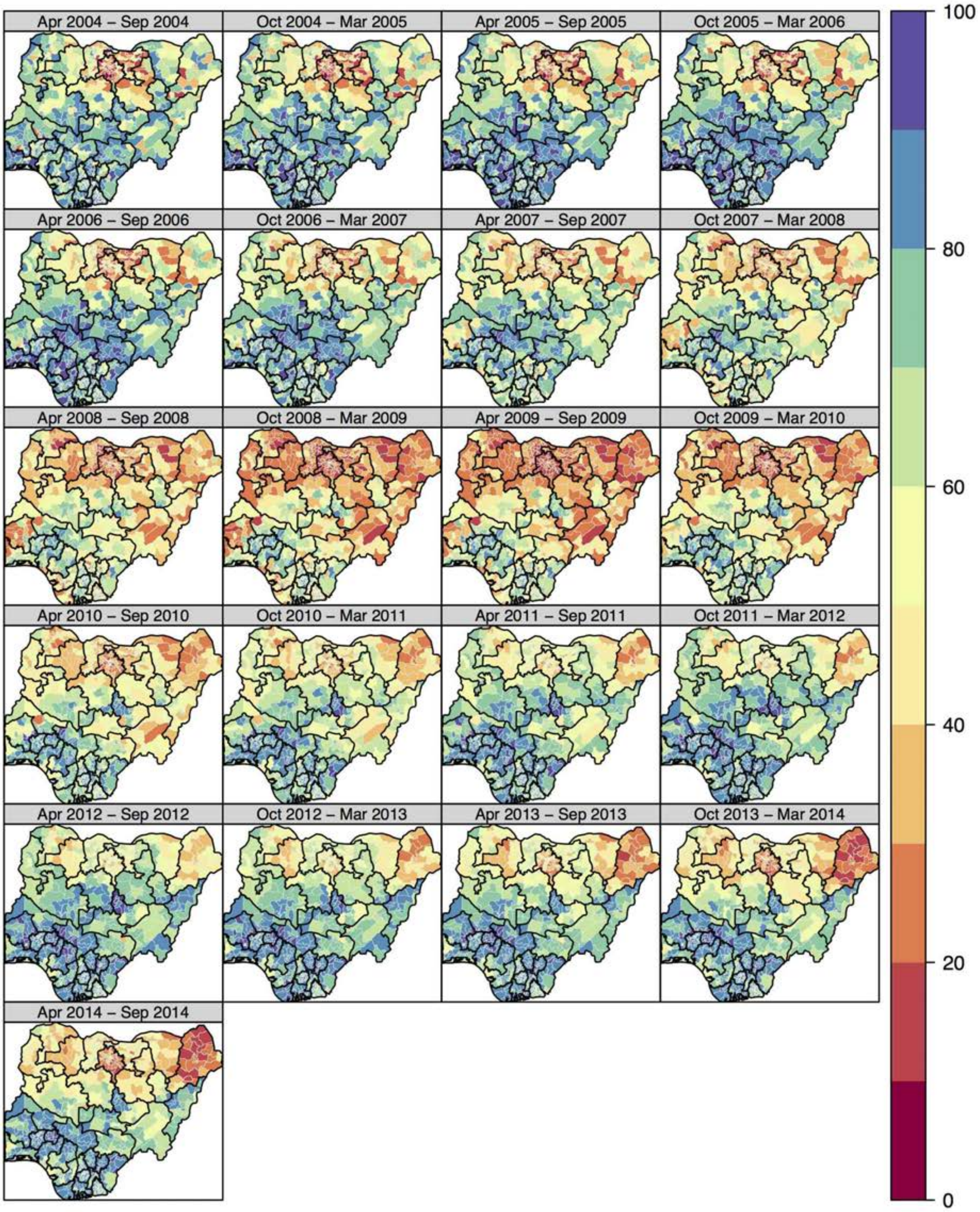

Figure H: Number of tOPV SIAs during each 6-month period. The publication of this map does not imply the expression of any opinion whatsoever on the part of WHO concerning the legal status of any territory, city or area or of its authorities, or concerning the delimitation of its frontiers or boundaries.

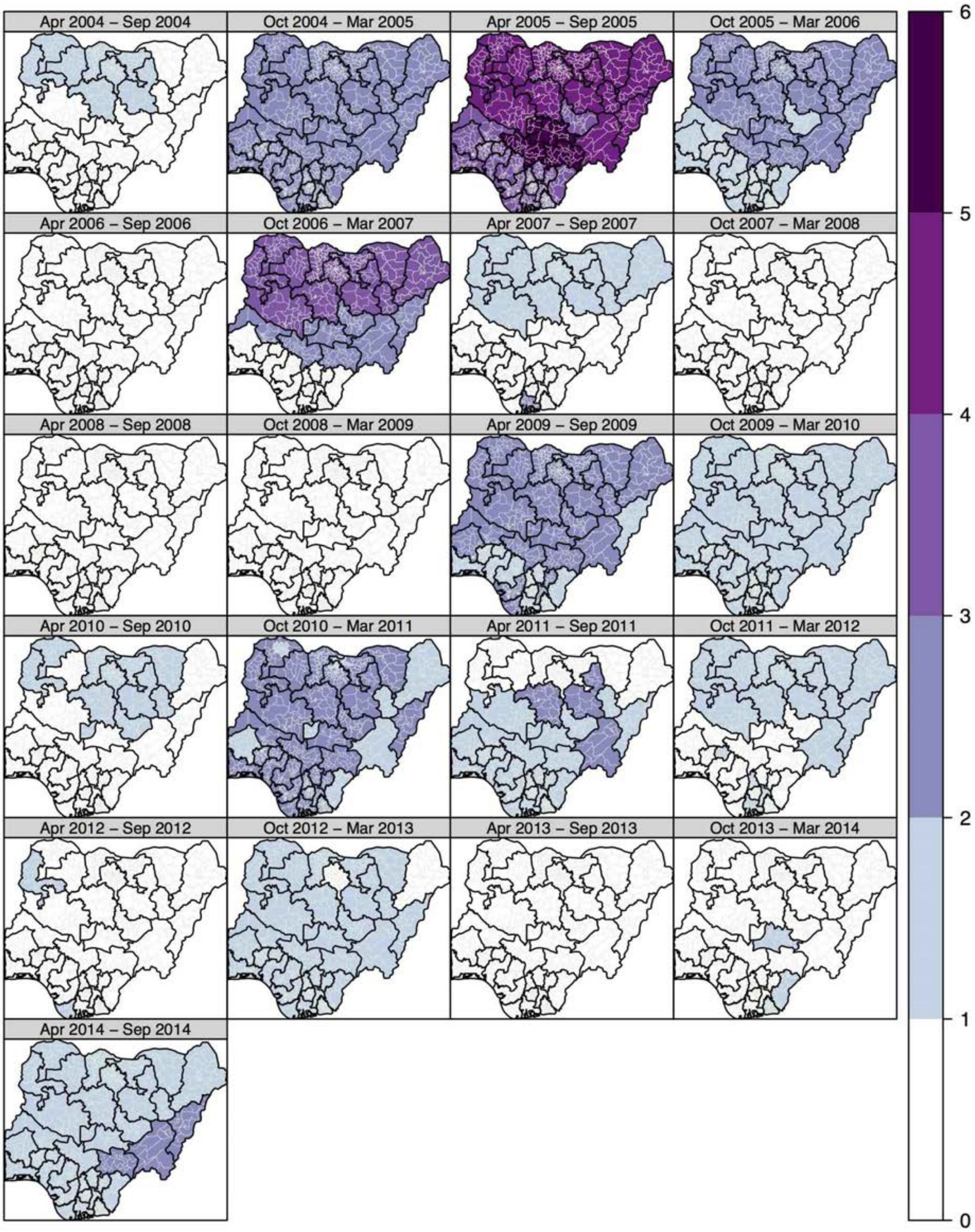

Figure I: Annual number of births per district (log10 scale) based on data for 2010 from WorldPop [11] and data on administrative boundaries in Nigeria. The publication of this map does not imply the expression of any opinion whatsoever on the part of WHO concerning the legal status of any territory, city or area or of its authorities, or concerning the delimitation of its frontiers or boundaries.

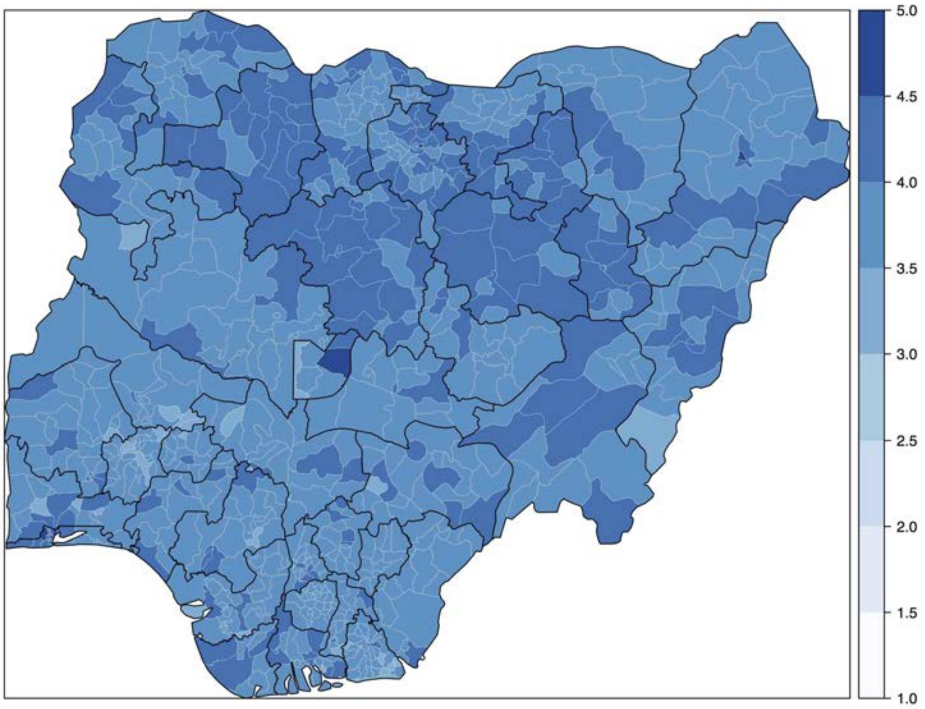

Figure J: Population density per district (log10 scale) using data for total population size and land size reported in the Census of 2006 [12]. The publication of this map does not imply the expression of any opinion whatsoever on the part of WHO concerning the legal status of any territory, city or area or of its authorities, or concerning the delimitation of its frontiers or boundaries.

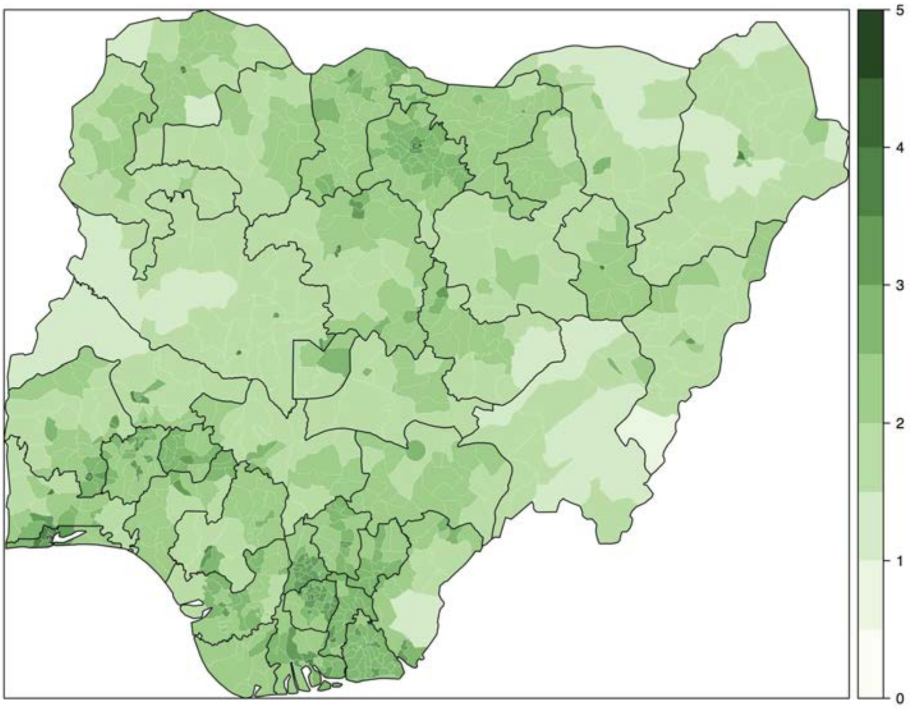

Figure K: Observed data in the DHS clusters (points) for the 2003, 2008 and 2013 DHS. (A) DTP3 coverage and (B) mean number of household members. The publication of this map does not imply the expression of any opinion whatsoever on the part of WHO concerning the legal status of any territory, city or area or of its authorities, or concerning the delimitation of its frontiers or boundaries.

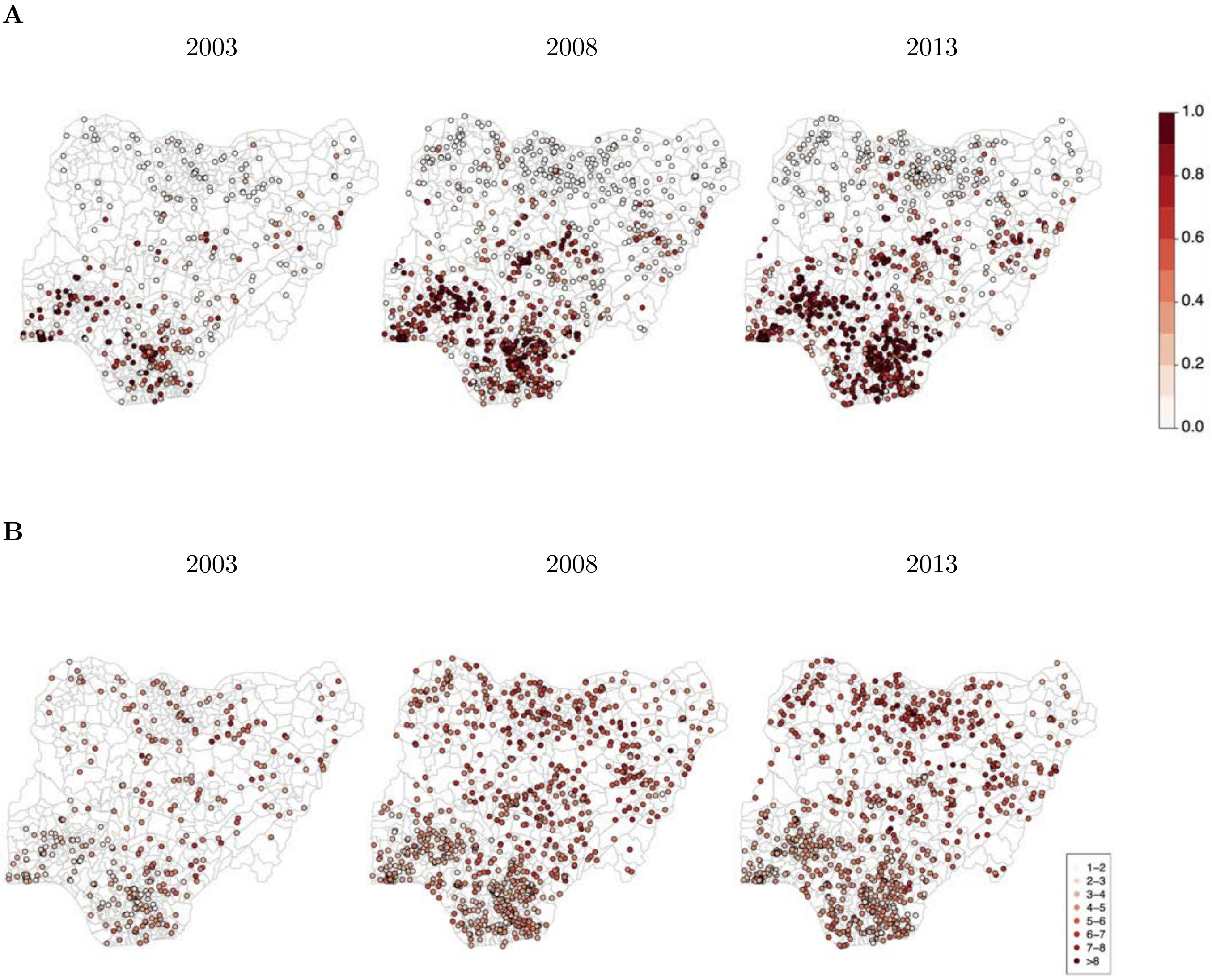

Figure L: Meshes used to obtain the GMRFs in the SPDE approach for the 2003, 2008 and 2013 DHS data for DTP3 coverage. Boundary of Nigeria (orange line), location of the clusters (red points), envelope containing all the clusters used to construct the mesh (blue line), and triangulation of the domain (black).

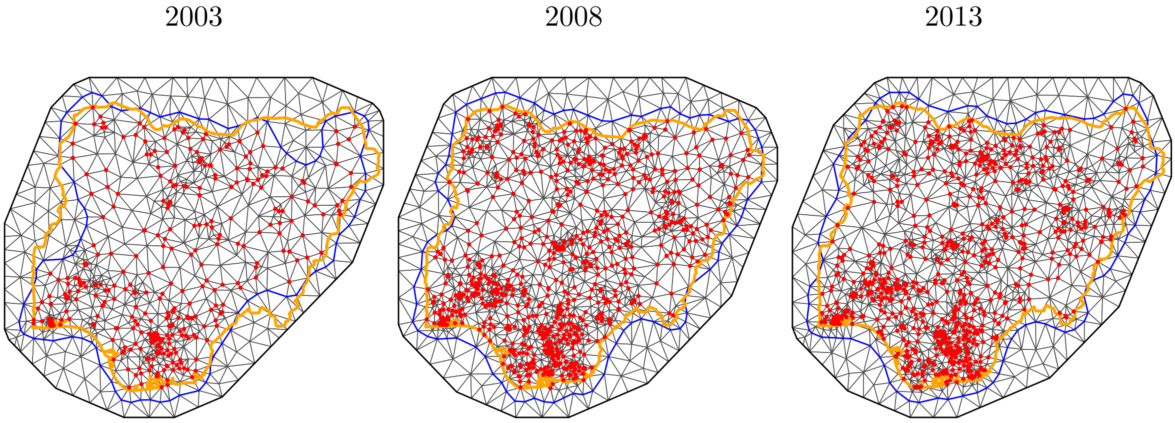

Figure M: Meshes used to obtain the GMRFs in the SPDE approach for the 2003, 2008 and 2013 DHS data for the mean number of household members. Boundary of Nigeria (orange line), location of the clusters (red points), envelope containing all the clusters used to construct the mesh (blue line), and triangulation of the domain (black).

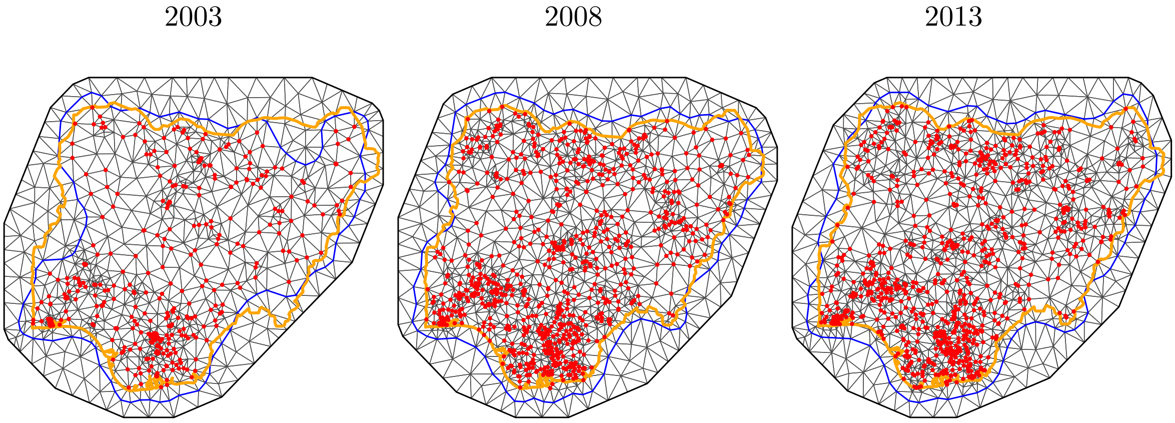

Figure N: Predictive performance of the spatial models for DHS data. Predictive performance statistics for the three models for DTP3 coverage (A, B), and for the three models for the mean number of household members (C, D). Boxplots of the Pearson correlation coefficient and Mean Squared Error were obtained using out-of-sample predictions with 10 random samples.

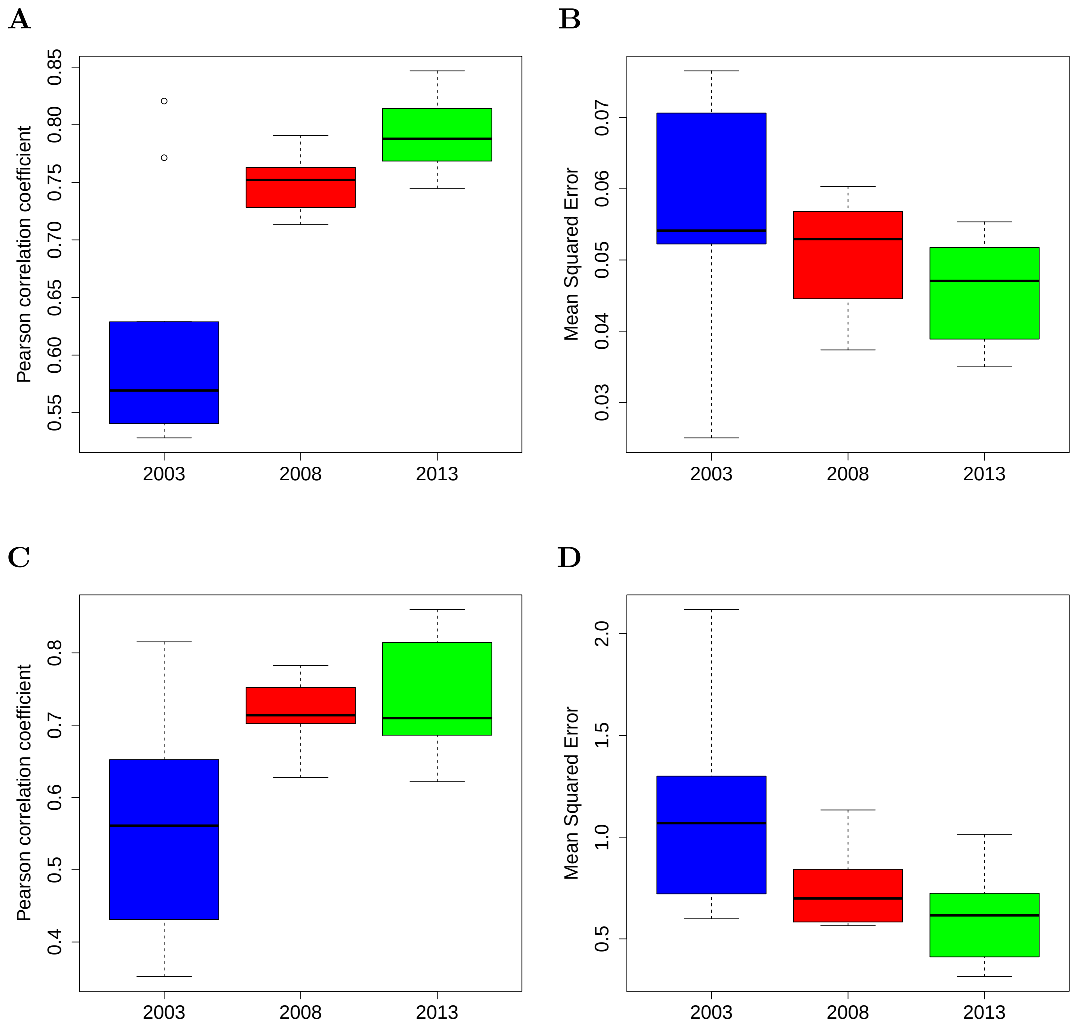

Figure O: Predicted DTP3 coverage. Maps of the predicted posterior mean (left) and standard deviation (right) of DTP3 coverage in Nigeria. Black crosses (+) represent the localisation of the DHS clusters (i.e. locations with observed data).

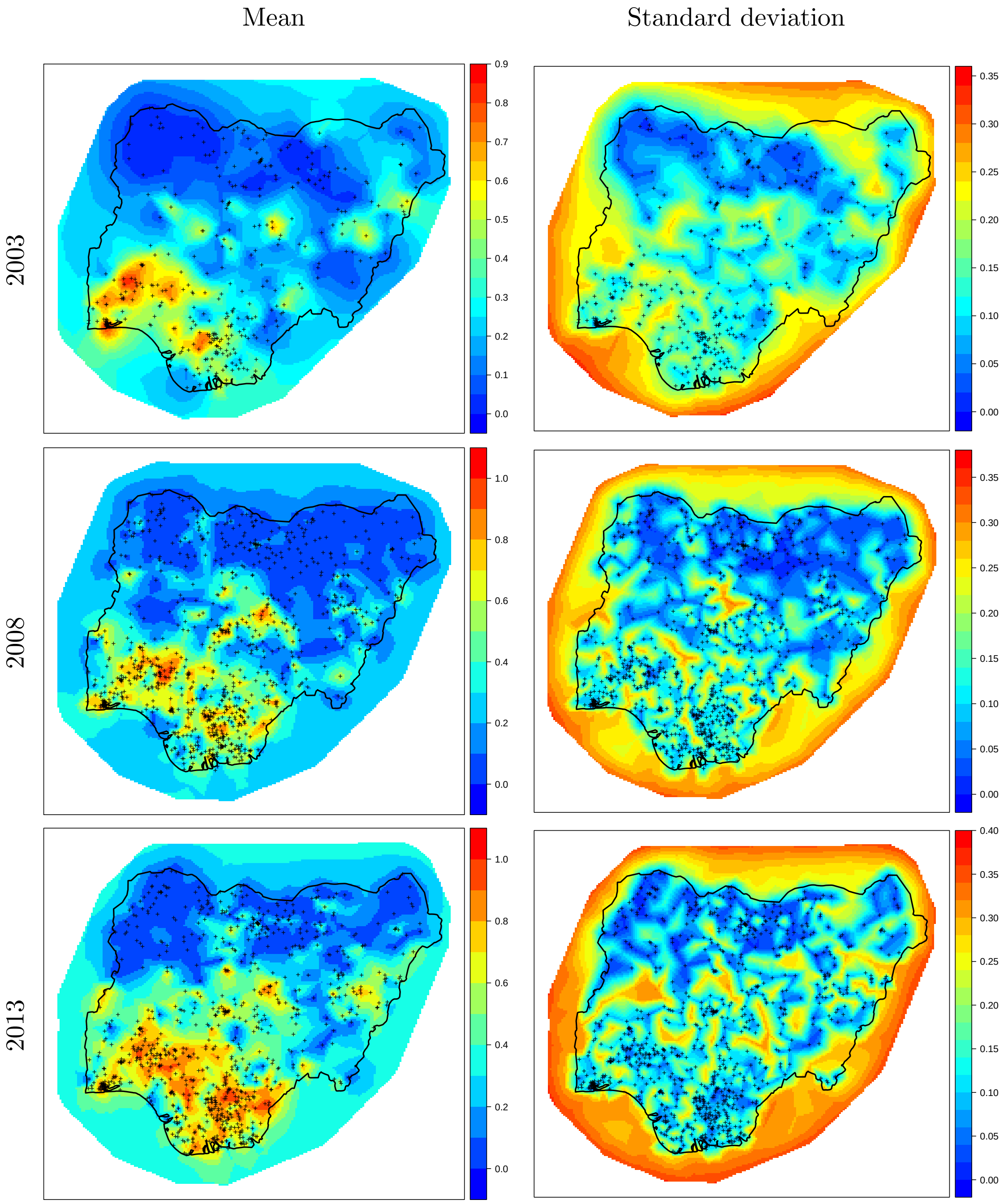

Figure P: Predicted number of household members. Maps of the predicted posterior mean (left) and standard deviation (right) of the mean number of household members in Nigeria. Black crosses (+) represent the localisation of the DHS clusters (i.e. locations with observed data).

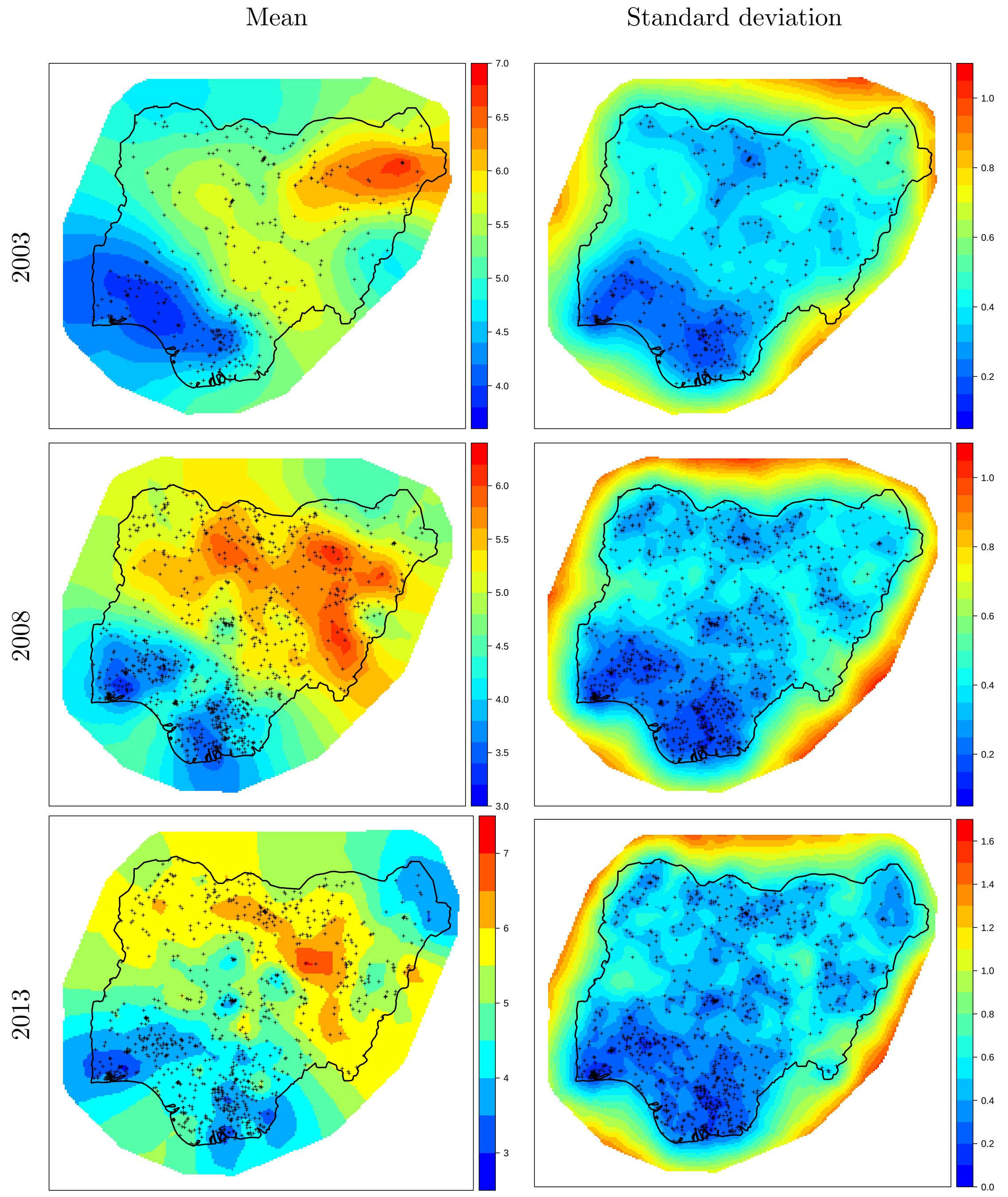

Figure Q: Predicted mean number of household members by district. The publication of this map does not imply the expression of any opinion whatsoever on the part of WHO concerning the legal status of any territory, city or area or of its authorities, or concerning the delimitation of its frontiers or boundaries.

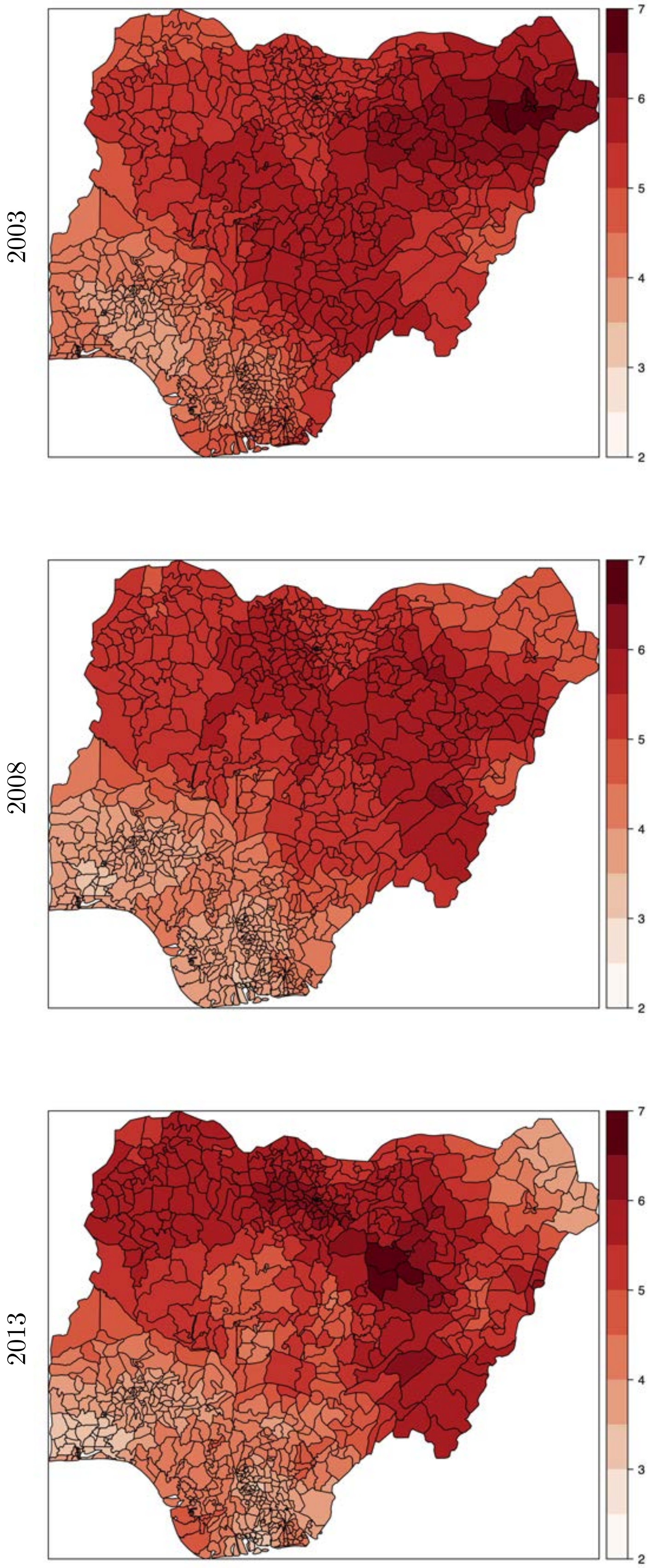

Table A: Description of the dynamic transmission model for the SIA random coverage hypothesis. List of events and their transitions. The variables are as follows: x is the proportion of susceptible, y is the proportion infected with Sabin virus, v is the proportion infected with a VDPV, and z is the proportion immune. The force of infection at time t for Sabin virus is given by $\lambda_S(t) = \beta_S y(t)$, and the force of infection for VDPVs is $\lambda_V(t) = \beta_V v(t)$.

Event	Transition	Rate
**Births - entering the system**		
Non-immunised through RI	$x \to x + 1$	$(1-c)\mu$
Immunised through RI without reversion to VDPV	$y \to y + 1$	$(1-\rho)c\mu$
Immunised through RI with reversion to VDPV	$v \to v + 1$	$\rho c\mu$
**Transmission of infections**		
Transmission of Sabin without reversion to VDPV	$y \to y + 1$	$(1-\rho)\lambda_S x$
	$x \to x - 1$	$(1-\rho)\lambda_S x$
Transmission of Sabin with reversion to VDPV	$v \to v + 1$	$\rho\lambda_S x$
	$x \to x - 1$	$\rho\lambda_S x$
Transmission of VDPV	$v \to v + 1$	$\lambda_V x$
	$x \to x - 1$	$\lambda_V x$
**Clearance of infections**		
Clearance of Sabin infections	$y \to y - 1$	γy
	$z \to z + 1$	γy
Clearance of VDPV infections	$v \to v - 1$	γv
	$z \to z + 1$	γv
**Leaving the system (at the age of 5)**		
	$x \to x - 1$	μx
	$y \to y - 1$	μy
	$v \to v - 1$	μv
	$z \to z - 1$	μz
**SIA**		
	$y \to y + (1-\rho)v_{sia}wx$	
	$v \to v + \rho v_{sia}wx$	
	$x \to x - v_{sia}wx$	

Table B: Description of the dynamic transmission model for the SIA fixed coverage hypothesis. List of events and their transitions. In this case, the population is structured into two groups, the "vaccinated" group (made up by all the individuals who receive OPV through SIAs), and the "missed" group. The variables are as follows: x_V (x_M) is the proportion of susceptible in the vaccinated (missed) group, y_V (y_M) is the proportion infected with Sabin virus in the vaccinated (missed) group, v_V (v_M) is the proportion infected with a VDPV in the vaccinated (missed) group, and z_V (z_M) is the proportion immune in the vaccinated (missed) group. The force of infection at time t for Sabin virus is given by $\lambda_S(t) = \beta_S(y_V(t) + y_M(t))$, and the force of infection for VDPVs is $\lambda_V(t) = \beta_V(v_V(t) + v_M(t))$.

Event	Transition	Rate
**Births - entering the system**		
Non-immunised through RI	$x_V \to x_V + 1$	$v_{sia}(1-c)\mu$
	$x_M \to x_M + 1$	$(1-v_{sia})(1-c)\mu$
Immunised through RI without reversion to VDPV	$y_V \to y_V + 1$	$v_{sia}(1-\rho)c\mu$
	$y_M \to y_M + 1$	$(1-v_{sia})(1-\rho)c\mu$
Immunised through RI with reversion to VDPV	$v_V \to v_V + 1$	$v_{sia}\rho c\mu$
	$v_M \to v_M + 1$	$(1-v_{sia})\rho c\mu$
**Transmission of infections**		
Transmission of Sabin without reversion to VDPV	$y_V \to y_V + 1$	$(1-\rho)\lambda_S x_V$
	$x_V \to x_V - 1$	$(1-\rho)\lambda_S x_V$
	$y_M \to y_M + 1$	$(1-\rho)\lambda_S x_M$
	$x_M \to x_M - 1$	$(1-\rho)\lambda_S x_M$
Transmission of Sabin with reversion to VDPV	$v_V \to v_V + 1$	$\rho\lambda_S x_V$
	$x_V \to x_V - 1$	$\rho\lambda_S x_V$
	$v_M \to v_M + 1$	$\rho\lambda_S x_M$
	$x_M \to x_M - 1$	$\rho\lambda_S x_M$
Transmission of VDPV	$v_V \to v_V + 1$	$\lambda_V x_V$
	$x_V \to x_V - 1$	$\lambda_V x_V$
	$v_M \to v_M + 1$	$\lambda_V x_M$
	$x_M \to x_M - 1$	$\lambda_V x_M$
**Clearance of infections**		
Clearance of Sabin infections	$y_V \to y_V - 1$	γy_V
	$z_V \to z_V + 1$	γy_V
	$y_M \to y_M - 1$	γy_M
	$z_M \to z_M + 1$	γy_M
Clearance of VDPV infections	$v_V \to v_V - 1$	γv_V
	$z_V \to z_V + 1$	γv_V
	$v_M \to v_M - 1$	γv_M
	$z_M \to z_M + 1$	γv_M
**Leaving the system (at the age of 5)**		
	$x_V \to x_V - 1$	μx_V
	$y_V \to y_V - 1$	μy_V
	$v_V \to v_V - 1$	μv_V
	$z_V \to z_V - 1$	μz_V
	$x_M \to x_M - 1$	μx_M
	$y_M \to y_M - 1$	μy_M
	$v_M \to v_M - 1$	μv_M
	$z_M \to z_M - 1$	μz_M
**SIA**		
	$y_V \to y_V + (1-\rho)wx_M$	
	$v_V \to v_V + \rho wx_M$	
	$x_V \to x_V - wx_V$	

Table C: Independent VDPV2 emergences in Nigeria (2004–2014). State, district and 6-month period where the first case of poliomyelitis associated with each of the 29 independent VDPV2 emergences were reported in Nigeria during 2004–2014. The last column indicates whether the isolate of the corresponding VDPV2 emergence established a circulating lineage (i.e. resulted in >1 case of poliomyelitis).

State	District	6-month period	Circulating lineage
BAUCHI	ITAS/GADAU	Apr 2005 – Sept 2005	Yes
LAGOS	MUSHIN	Apr 2005 – Sept 2005	No
SOKOTO	ILLELA	Apr 2005 – Sept 2005	No
KADUNA	SABON GARI	Oct 2005 – Mar 2006	No
KEBBI	KOKO/BESSE	Oct 2005 – Mar 2006	No
JIGAWA	KIYAWA	Apr 2006 – Sept 2006	Yes
BORNO	KONDUGA	Apr 2006 – Sept 2006	No
BORNO	MAIDUGURI	Apr 2006 – Sept 2006	No
JIGAWA	YANKWASHI	Apr 2006 – Sept 2006	No
BORNO	MAIDUGURI	Apr 2006 – Sept 2006	Yes
JIGAWA	GARKI	Apr 2006 – Sept 2006	Yes
BAUCHI	ZAKI	Oct 2006 – Mar 2007	No
SOKOTO	GADA	Oct 2006 – Mar 2007	Yes
NIGER	WUSHISHI	Apr 2007 – Sept 2007	No
ANAMBRA	IDEMILI SOUTH	Oct 2007 – Mar 2008	No
BAUCHI	DARAZO	Apr 2008 – Sept 2008	No
SOKOTO	BODINGA	Apr 2008 – Sept 2008	No
KEBBI	SURU	Oct 2008 – Mar 2009	Yes
KATSINA	DAURA	Oct 2008 – Mar 2009	No
BORNO	DAMBOA	Apr 2009 – Sept 2009	Yes
KADUNA	ZARIA	Apr 2009 – Sept 2009	No
KEBBI	ALEIRO	Oct 2010 – Mar 2011	No
NIGER	BIDA	Oct 2011 – Mar 2012	No
EDO	OVIA SOUTH-WEST	Apr 2012 – Sept 2012	No
KEBBI	KOKO/BESSE	Oct 2012 – Mar 2013	No
GOMBE	DUKKU	Apr 2013 – Sept 2013	No
CROSS RIVER	CALABAR SOUTH	Oct 2013 – Mar 2014	No
FCT, ABUJA	BWARI	Apr 2014 – Sept 2014	No
KANO	KURA	Apr 2014 – Sept 2014	No

Table D: Parameter estimates (mean and 95% credible interval) for the spatio-temporal random-effects model of serotype-2 population immunity.

Parameters	Mean (95% credible interval)
Intercept, α	0.298 (0.159 – 0.436)
Precision for the Gaussian observations, $1/\sigma^2$	37.683 (37.673 – 37.701)
Precision for the district-temporal interaction, $1/\sigma_\gamma^2$	110.005 (109.974 – 113.258)
Precision for the state-temporal interaction, $1/\sigma_\delta^2$	35.498 (35.483 – 35.524)

Table E: Number of DHS clusters. Number of clusters (observations) for the three DHS datasets (2003, 2008, 2013), and number of triangles in the mesh used for SPDE to predict the DTP3 coverage (left) and the mean number of household members (right).

DTP3 coverage	2003	2008	2013
No. clusters	357	886	889
No. triangles (mesh)	755	1327	1374

House members	2003	2008	2013
No. clusters	360	886	889
No. triangles (mesh)	755	1327	1374

Table F: Parameter estimates for the models of DTP3 coverage. Posterior estimates (mean and 95% credible interval) of model parameters for the three spatial models for DTP3 coverage (2003, 2008, 2013).

Parameters	2003	2008	2013
Intercept, α	-1.935 (-2.641 – -1.300)	-1.886 (-2.319 – -1.469)	-1.256 (-1.735 – -0.792)
Nominal variance, σ	2.840 (1.897 – 4.140)	3.987 (3.285 – 4.821)	4.762 (3.902 – 5.775)
Nominal range, ρ (km)	149 (101 – 213)	83 (71 – 97)	84 (72 – 97)

Table G: Parameter estimates for the models of the number of household members. Posterior estimates (mean and 95% credible interval) of model parameters for the three spatial models for the mean number of household members (2003, 2008, 2013).

Parameters	2003	2008	2013
Precision of Gamma, ϕ	30.749 (26.083 – 35.860)	32.103 (28.641 – 35.784)	38.888 (34.457 – 43.673)
Intercept, α	1.625 (1.406 – 1.843)	1.572 (1.453 – 1.689)	1.580 (1.482 – 1.679)
Nominal variance, σ	0.021 (0.009 – 0.045)	0.021 (0.012 – 0.035)	0.032 (0.021 – 0.047)
Nominal range (km), ρ	560 (289 – 1036)	361 (223 – 580)	241 (172 – 333)

References

[1] Farrington, C. P. & Grant, A. D. The distribution of time to extinction in subcritical branching processes: applications to outbreaks of infectious disease. *Journal of Applied Probability* **36**, 771–779 (1999).

[2] Blake, I. M. *et al.* The role of older children and adults in wild poliovirus transmission. *Proceedings of the National Academy of Sciences* **111**, 10604–10609 (2014).

[3] Casey, A. E. Observations on an Epidemic of Poliomyelitis. *Science* **95**, 359–360 (1942).

[4] Alexander, J. P., Gary, H. E. & Pallansch, M. A. Duration of poliovirus excretion and its implications for acute flaccid paralysis surveillance: a review of the literature. *Journal of Infectious Diseases* **175**, S176–S182 (1997).

[5] Mangal, T. D. *et al.* Key issues in the persistence of poliomyelitis in nigeria: a case-control study. *The Lancet Global Health* **2**, e90–e97 (2014).

[6] Grassly, N. C. *et al.* New strategies for the elimination of polio from India. *Science* **314**, 1150–1153 (2006).

[7] ICF International. Demographic and Health Surveys (various) [Nigeria 2003: NGKR4BFL.SAV, NGHR4BFL.SAV, NGGE4BFL; Nigeria 2008: NGKR52FL.SAV, NGHR52FL.SAV, NGGE52FL; Nigeria 2013: NGKR6AFL.SAV, NGHR6AFL.SAV, NGGE6AFL]. Calverton, Maryland: ICF International.

[8] Lindgren, F., Rue, H. & Lindström, J. An explicit link between Gaussian fields and Gaussian Markov random fields: the stochastic partial differential equation approach. *Journal of the Royal Statistical Society: Series B* **73**, 423–498 (2011).

[9] Rue, H., Martino, S. & Chopin, N. Approximate Bayesian inference for latent Gaussian models by using integrated nested Laplace approximations. *Journal of the Royal Statistical Society: Series B* **71**, 319–392 (2009).

[10] R-INLA. URL `www.r-inla.org`.

[11] WorldPop Project. URL `www.worldpop.org.uk`.

[12] 2006 Population and Housing Census. Population distribution by sex, state, LGA and Senatorial District. Volume 3. National Population Commission of the Federal Republic of Nigeria (2010).

[13] Knorr-Held, L. Bayesian modelling of inseparable space-time variation in disease risk. *Statistics in Medicine* **19**, 2555–2567 (2000).

[14] Upfill-Brown, A. M. *et al.* Predictive spatial risk model of poliovirus to aid prioritization and hasten eradication in Nigeria. *BMC Medicine* **12**, 92 (2014).

[15] Lindgren, F. & Rue, H. Bayesian spatial modelling with R-INLA. *Journal of Statistical Software* **19** (2015).
